# Detection of porphyrins in vertebrate fossils from the Messel and implications for organic preservation in the fossil record

**DOI:** 10.1371/journal.pone.0269568

**Published:** 2022-06-29

**Authors:** Sandra Siljeström, Anna Neubeck, Andrew Steele

**Affiliations:** 1 Department of Methodology, Textiles and Medical Technology, RISE Research Institutes of Sweden, Stockholm, Sweden; 2 Department of Earth Sciences, Uppsala University, Uppsala, Sweden; 3 Carnegie Institution for Science, Earth and Planetary Laboratory, Washington, DC, United States of America; University of Paris, FRANCE

## Abstract

Organic molecules preserved in fossils provide a wealth of new information about ancient life. The discovery of almost unaltered complex organic molecules in well-preserved fossils raise the question of how common such occurrences are in the fossil record, how to differentiate between endogenous and exogenous sources for the organic matter and what promotes such preservation. The aim of this study was the in-situ analysis of a well-preserved vertebrate fossil from 48 Ma Eocene sediments in the Messel pit, Germany for preservation of complex biomolecules. The fossil was characterized using a variety of techniques including time-of-flight secondary ion mass spectrometry (ToF-SIMS), scanning electron microscopy/energy dispersive x-ray spectroscopy (SEM/EDX), x-ray diffraction (XRD) and Raman spectroscopy. A suite of organic molecules was detected, including porphyrins, which given the context of the detected signal are most probably diagenetically altered heme originating from the fossil though a microbial contribution cannot be completely ruled out. Diagenetic changes to the porphyrin structure were observed that included the exchange of the central iron by nickel. Further analyses on the geochemistry of the fossil and surrounding sediments showed presence of pyrite and aluminosilicates, most likely clay. In addition, a carbonate and calcium phosphate dominated crust has formed around the fossil. This suggests that several different processes are involved in the preservation of the fossil and the organic molecules associated with it. Similar processes seem to have also been involved in preservation of heme in fossils from other localities.

## Introduction

The Eocene (~48 Ma) sediments in the Messel Pit, Germany, is known for its exceptional preservation of a diversity of fossils, including mammals, fish, plants and insects [1, 2]. The fossils are often preserved in exquisite detail, including preservation of soft tissues, such as feathers, skin, stomach contents and melanosomes [1–3]. Recently it has also been shown that other soft-tissues such as osteocytes and blood vessel-like structures in bones, and an uropygial gland of a bird are also preserved [4, 5]. In addition, Messel is known to preserve almost unaltered organic molecules both in association with fossils and in sediments containing the fossils [4, 6–10]. In order to get a better understanding of the origin and preservation of organic soft tissue and molecules in the fossil record including Messel, subsamples of an exceptionally preserved vertebrate fossil from Messel were analysed in a spatially resolved fashion with time-of-flight secondary ion mass spectrometry (ToF-SIMS). The ToF-SIMS data was then compared with data from other techniques such as microscopy, scanning electron microscopy/ energy dispersive x-ray spectroscopy (SEM/EDX), Raman spectroscopy, and x-ray diffraction (XRD) to better understand the preservation environment of the organic molecules detected.

## Materials and methods

### Field collection of samples

Samples were collected in 2003 at the Messel Pit (Grube Messel), a Konservat Lagerstätte, (near Darmstadt, Germany) by permission of the Senckenberg Research Institute (SNG), the division of Messel Research (B. Behr, M. Felder and F.-J Harms) to Maia K. Schweizer [6]. All necessary permits were obtained for the described study, which complied with all relevant regulations.

The collected samples were obtained from about 1m above the so called alpha horizon of the Messel Fossillagerstätte (Messel oil shale, Middle Messel Formation) [6, 11]. Only well-preserved fossils with soft tissue or sub-samples of these samples were collected for study. In order to minimize organic contamination, the samples were collected in the field with cleaned and sterilized micro-picks and wrapped in organic-clean aluminum foil (muffled to 500°C) [6]. The samples were refrigerated in the field to prevent microbial contamination/ degradation of the fossils and then continuously kept frozen at -20°C until ToF-SIMS analyses.

### Samples

The following fossils and sediments from the Messel Pit were studied.

**Fig 1:**
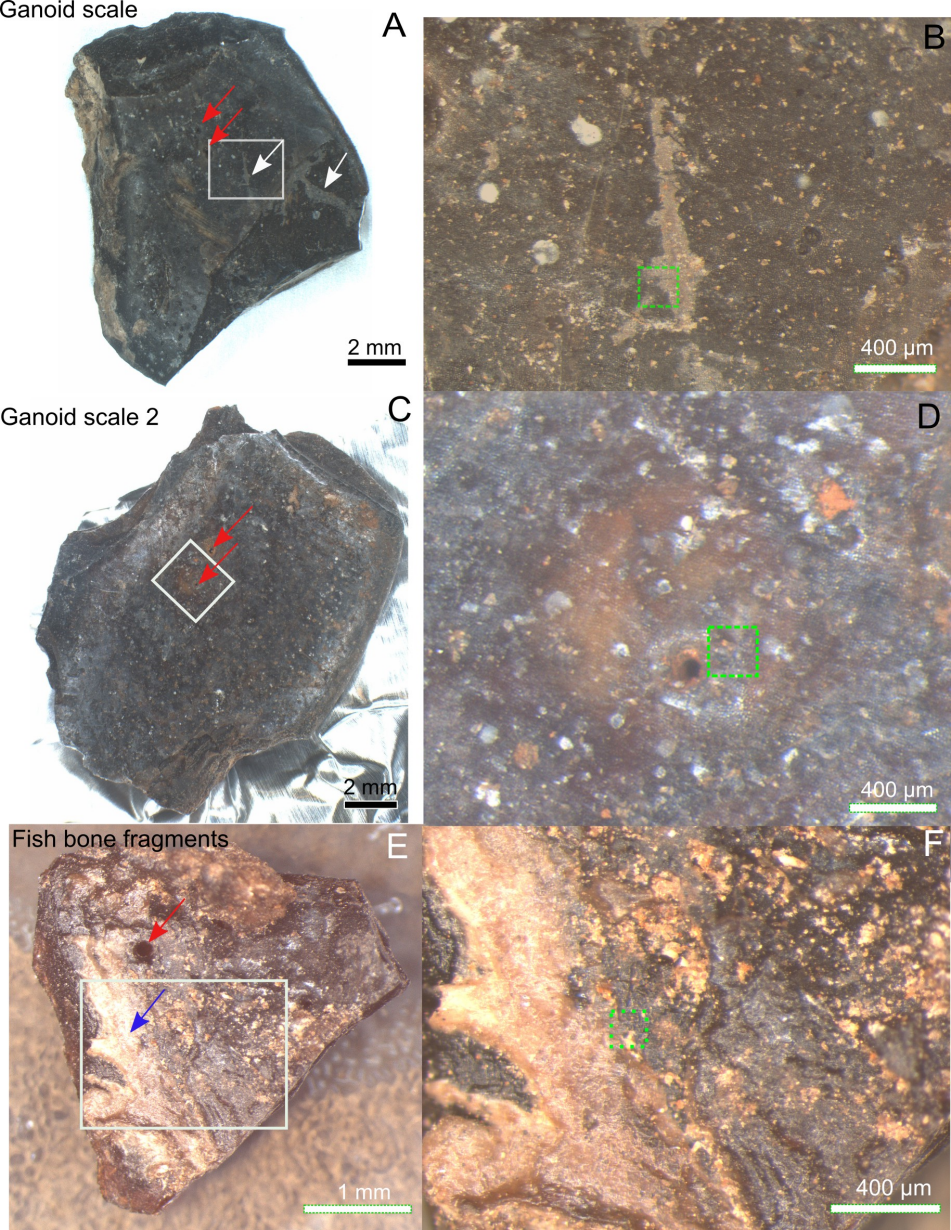
Messel fish *Atractosteus* ganoid scales and bones where porphyrins were detected. A) Ganoid scale 1 with gray-brown film. White arrows indicate presence of gray-brown film on parts of ganoid layer and exposed laminar bone. Red arrow shows aperture penetrating ganoid layer. B) Zoom-in of white box in A. C) Ganoid scale 2. Red arrows indicate presence of apertures in ganoid layer. D) Zoom-in of white box in C. E) Fish bone fragment. Red arrow indicates foramen in bone while blue arrow indicate area with whitish non-bony structure. F) Zoom-in of white box in E. Green dotted squares in B, D, F indicates precise area of ToF-SIMS analysis in Figs 2 and 3 and S3–S5.

- Subsamples from a well-preserved fish fossil (*Atractosteus*, original fossil was appr. 80 cm long, MEMS201) including ganoid scales, bone fragments and a vertebra, and sediment attached to the fossils (Figs 1 and S1A–S1H) [6, 11, 12].
- A piece of the Messel oil shale (MEMS17) collected within 1m of fish fossil but never in direct contact with fossil (S1I Fig). The piece contains a snail fossil (S1J Fig) and was studied as a representative of the general mineralogy of the Messel shale.

The studied samples (MEMS 201 and MEMS17) are permanently stored under their field names in a freezer at the Earth and Planetary Laboratory at Carnegie Institution of Science in Washington DC, USA as agreed with the Senckenberg Research Institute (SNG), the division of Messel Research. All specimens are fully available for research.

Additionally, standards of heme b (pig hemoglobin) and chlorophyll *a* from SigmaAldrich were analysed. Data was also compared with data collected previously on a fossil mosquito with preserved heme in the abdomen, from the Kishenehn formation, Montanta, USA [13].

### Microscopy and scanning electron microscope (SEM)/electron-dispersive x-ray spectroscopy (EDX)

Micrographs were obtained by use of an Olympus stereo microscope (SZX16) and Nikon (Optihop 100S) in reflective mode with 20x and 50x LWD objectives. Fluorescence micrographs were obtained by an Olympus BX-63 epifluorescence microscope equipped with X-Cite 120Q Microscope Illumination System with a 120 W Mercury Vapor Short Arc lamp, a 360–370 nm, bandpass excitation filter, and a 420 nm longpass emission filter.

SEM images were obtained after ToF-SIMS analysis. The samples were imaged uncoated in a Supra 40 VP FEG SEM (Zeiss, Germany) at high vacuum (7 x 10^−7^ mbar) operating at 1–2 keV in secondary electron mode. EDX data was obtained with an Oxford Instruments Xmax^50^ (50mm2 SSD Detector) at 17keV in low vacuum (3.8 x 10^−4^ mbar) secondary electron mode.

### Time-of-flight secondary ion mass spectrometry (ToF-SIMS)

ToF-SIMS scans a surface with a high energy ion beam. In addition to mass spectra, ion images are also obtained. The chemical data in these ion images can be matched with morphological structures in optical and electron micrographs. This feature helps in determining whether organic molecules are indigenous to fossil material rather than having been later introduction by bacteria or other contamination.

The samples were taken out from the freezer just before analysis. The samples were mounted directly on the ToF-SIMS sample holder either by clamps or placed on double-sided sticky tape on a Si wafer, using cleaned tweezers (heptane, acetone and ethanol, in that order) in a laminar flow hood. After mounting of the samples, the sample holder was directly introduced into the ToF-SIMS.

Analysis of fossils were performed in a ToF-SIMS IV instrument (ION-TOF GmbH, Germany) located at RISE Research Institute of Sweden in Borås, Sweden. Samples were analysed by rastering a 25keV Bi_3_^+^ beam over an 100x100 to 500x500 μm^2^ area for 200–500 s. The analyses were performed in both positive and negative mode at high mass resolution (bunched mode: m/Δm ≥ 5000 at m/z 30, Δl ~ 5μm) with a pulsed current of 0.1 pA. In addition, spectra were collected at high spatial resolution (burst alignment mode: m/Δm ~100–300, Δl < 1 μm) with a pulsed current of ⁓0.045 pA. As the samples were insulating, the sample surface was flooded with electrons for charge compensation.

The assignments of the peaks in the ToF-SIMS spectra were based on comparisons with the spectra of standards such as heme b, chlorophyll a and previously published literature [13–16].

### Raman spectroscopy

Due to the high fluorescence exhibited by the sample, Raman spectroscopy were performed on two different systems; a RM 100, Renishaw equipped with a blue laser and a Witec α-scanning near-field optical microscope equipped with a green laser. The green Raman system allowed us to map the organic signal while the blue Raman system allowed us to detect mineral signals not observed by the green Raman.

#### RM 1000, Renishaw (blue laser, 465 nm)

Raman spectra were collected on a confocal Raman microscope (RM 1000, Renishaw) using a 465 nm doubled Nd: YAG laser with 0.1–5 mW laser power and an 1800 lines/mm grating. Acquisition time for each spectrum was 10 s from 100 to 2000 cm^-1^. All spectral peaks were calibrated against the value of 520.7 cm^-1^ of a silicon wafer.

Assignments of mineral and organic peaks in the spectra were done by comparison to spectra in the RRUFF database and literature [17–22].

#### Witec α-scanning near-field optical microscope (green laser, 532nm)

Raman spectra and images were collected using a Witec α-scanning near-field optical microscope that has been customized to incorporate confocal Raman spectroscopic imaging [23, 24]. The excitation source is a frequency-doubled solid-state YAG laser (532nm) operating between 0.3 and 1 mW output power (dependent on objective), as measured at the sample using a laser power meter [23, 24]. Objective lenses used included a x100 super long working distance (SLWD) and a x20 long working distance (LWD) with a 20 μm optical fiber acting as the confocal pin hole. Spectra were collected on a Peltier-cooled Andor EMCCD chip, after passing through a f/4 300mm focal length imaging spectrometer typically using a 600 lines/mm grating [23]. The lateral resolution of the instrument is as small as 360 nm in air when using the x100 SLWD objective, with a focal plane depth of ~800nm [23].

This instrument can operate in several modes. Typically, 2D imaging and single spectra modes were used during this study. Single spectra mode allows the acquisition of a spectrum from a single spot on the target. Average spectra are produced typically using integration times of 30 seconds per accumulation and 10 accumulations to allow verification of weak spectral features [23]. For the images the instrument then takes a Raman spectrum (0–3600 cm^-1^ using the 600 lines mm^-1^ grating) at each pixel using an integration time of between 1 and 6 s per pixel [23].

### X-ray diffraction (XRD)

The materials were carefully grinded and smeared onto a Zero Background Holder (ZBH) with a spatula. The ZBH was then mounted in a stainless-steel holder and put in the X-Ray Powder Diffractometer. The measurements were performed at room temperature (approximately 22°C) on a PANalytical X’Pert PRO diffractometer, equipped with a Cu, long fine focus X-ray tube and a PIXcel detector [25]. Automatic divergence- and anti-scatter slits were used together with 0.02 rad soller slits and a Ni-filter. The scan length was approximately 65 minutes. In order to increase the randomness of the samples they were spun during the analysis [25]. The samples were analyzed between 2–80° in 2-theta using 255 detector channels [26]. The recorded data was evaluated using the X´Pert HighScore software [27]. A peak search was performed followed by a search-match against a the ICDD PDF2 reference database to identify the crystalline part of the material.

## Results

### Stereomicroscopy of fossils, sediments and shale collected nearby

Dark black/brown (1–30 mm) bone fragments, ganoid scales and one vertebra of the *Atractosteus* fossil were studied (Figs 1 and S1). Reddish white-brown sediment (S1E Fig) was attached to the fossil (Figs 1 and S1A–S1H), especially at the base of one of the scales (S1D Fig) and, on the bone fragments (Figs 1E and 1F and S1B and S1H) and the vertebra (S1C Fig). Cavities, sometimes partly filled with the sediment, were found throughout the fossil (Figs 1 and S1A–S1H).

A gray-brown elongated film covered part of the crown surface of one of the scales (white arrows, Fig 1A and 1B), and was only associated with one of the four most morphologically intact scales and additional scale fragments studied. The film extended from the ganoid surface of the scale to parts of the scale where the ganoid layer had been removed, exposing the basal plate (lamellar bone) of the scale, suggesting that it was deposited after the scale broke apart. This particular scale was the only example where the ganoid layer had been removed to expose the laminar bone [28]. The scales’ ganoid surfaces were ornamented with tubercles and pierced by apertures (possibly Williamson canals) which sometimes appeared to penetrate the whole ganoid layer to the laminar bone (red arrows in Figs 1A–1D and S1F and S1G) [28]. These apertures were sometimes partly filled by the reddish white-brown sediment and occasionally surrounded by a halo of reddish material. Some bone fragments had a whitish non-bony structure protruding from them (Fig 1E and 1F, blue arrow). Also at least one of the bone fragments has what appeared to be a foramen piercing the bone (Fig 1E, red arrow). The microscopy of the fossils showed that, despite being frozen after collection, some fungal hyphae were present on the piece of Messel oil shale (S1I–S1K Fig). However, no hyphae were found on the fish fossil (Figs 1 and S1).

Four scales, two different bone fragments and one vertebra of the *Atractosteus* fossil were studied by ToF-SIMS and reflective microscopy (Figs 1 and S1C and S1F–S1H). From the ToF-SIMS results one of the scales and one of the bone fragments were then chosen for further studies by SEM/EDX and Raman spectroscopy (Fig 1A and 1E). Reddish white-brown sediment attached to the fossil and a piece of the Messel oil shale collected near fossils were studied with ToF-SIMS, SEM/EDX, Raman spectroscopy and XRD (S1C–S1E and S1I Fig).

### Organic signals from fossil, sediment and shale

**Fig 2:**
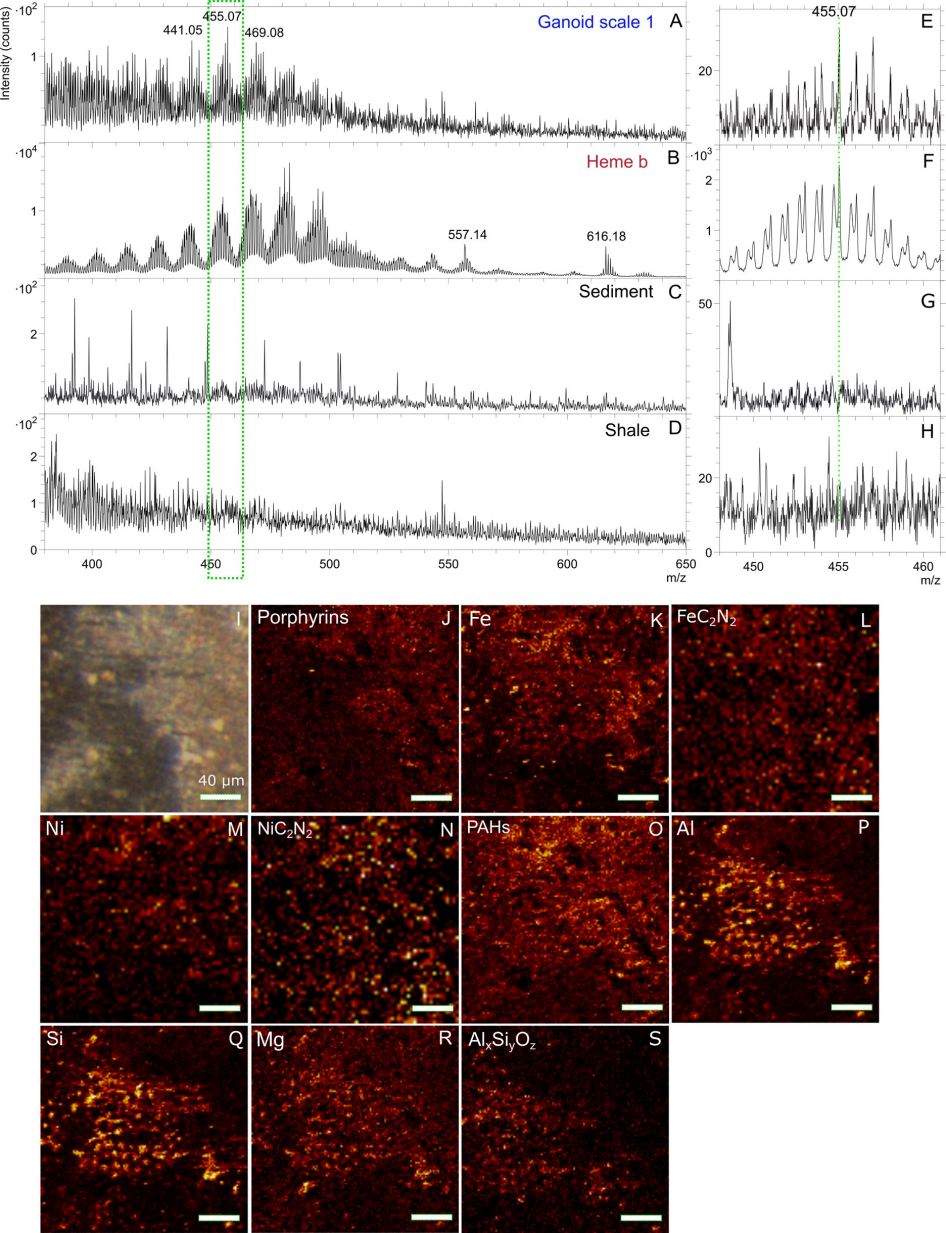
Molecular characterization of gray-brown film on Messel fossil fish scale by ToF-SIMS indicating porphyrins. Positive ToF-SIMS spectra (m/z 380–650) of A) area indicated by green dotted box on ganoid scale 1 in Fig 1B, B) heme b standard, C) sediment attached to scale (S1 Fig) and D) shale sample collected nearby (S1 Fig). E-H) Zoom-in of green dotted box in spectra in A-D showing m/z 448–461. I) Micrograph of area in green dotted box in Fig 1B. High spatial resolution ToF-SIMS ion images of J) sum of porphyrin peaks (m/z 434–447, 449–461, 462–473 and 474–485), K) Fe^+^, L) FeC_2_N_2_^-^, M) Ni^+^, N) NiC_2_N_2_^-^, O) sum of PAH:s (m/z 77, 91, 139, 141 and 165), P) Al^+^, Q) Si^+^, R) Mg^+^ and S) sum of AlSiO_4_^-^ and AlSi_2_O_6_^-^.

**Fig 3:**
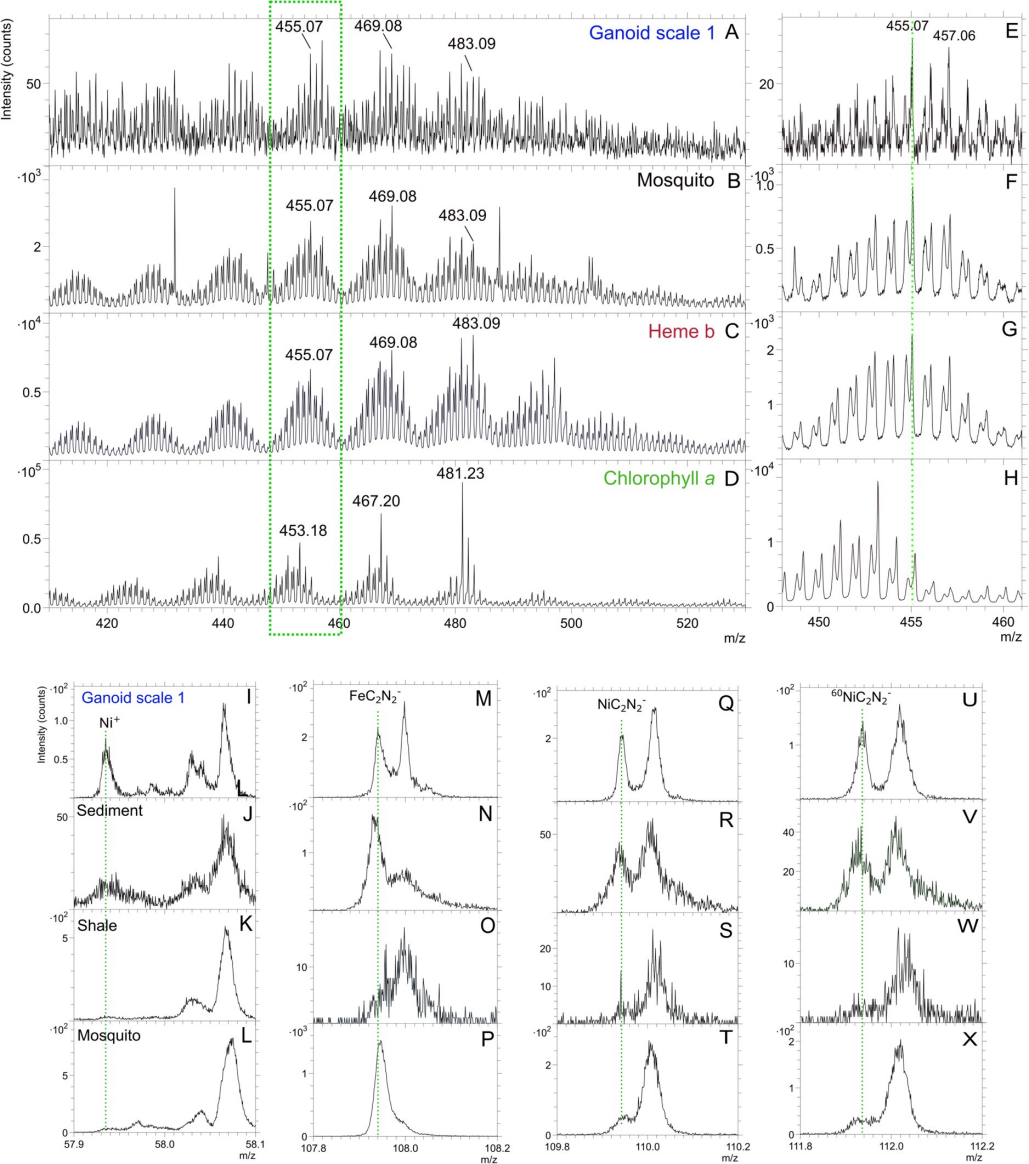
Molecular characterization of gray-brown film on Messel fossil scale by ToF-SIMS indicating diagenetically altered porphyrins. Positive ToF-SIMS spectra (m/z 410–530) of A) area indicated by green dotted box on ganoid scale 1 in Fig 1B, B) mosquito, C) heme b standard and D) chlorophyll *a* standard. E-H) Zoom-in of green dotted box in spectra in A-D showing m/z 448–461. ToF-SIMS spectra of fish scale with gray-brown film (I, M, Q, U), sediment attached to scale (J, N, R, V), shale sample collected nearby (K, O, S, W) and mosquito (L, P, T, X) showing Ni^+^ (m/z 57.94), FeC_2_N_2_^-^ (m/z 107.94), NiC_2_N_2_^-^ (m/z 109.94) and ^60^NiC_2_N_2_^-^ (m/z 111.94).

In positive ToF-SIMS analysis of scale 1 (Figs 1B, 2, 3 and S3) a clear distribution of peaks, in clusters centering around m/z 441.05, 455.07, 469.08 and 483.09 (Fig 2A and 2E and S1 Table) were found. These peaks showed a Gaussian distribution, where the strongest peak in each peak cluster was separated by m/z 14 (CH_2_). This peak patterning has been shown to be typical and indicative of porphyrins [13, 16, 29, 30]. The highest signal intensities of the peaks (m/z 441, 455, 469 and 483) were obtained in spectra collected from scale 1 and ToF-SIMS ion images of these peaks showed that they mostly localized to the gray-brown film on the scale (Fig 2I and 2J). Similar peak patterns were also found in spectra collected on a bone fragment (Figs 1F and S3B and S3E, S4A and S4B) and in spectra from scale 2 near one of the pores (Williamson canals) of that scale (Figs 1D and S3C and S3F, S5A and S5B). This pattern of peaks was only present in 1 out 6 analyses of the sediment attached to the fossil (Figs 2C and 2G and S1D and S1E) and none of the 10 analyses of the nearby collected shale sample (Figs 2D and 2H and S1I). Additionally, associated with porphyrin signal on the fossil are peaks in the negative ion spectra that can be assigned to FeC_2_N_2_^-^, NiC_2_N_2_^-^_,_ and ^60^NiC_2_N_2_ (Figs 2 and 3 and S1 Table). These peaks were also present in the spectra of the sediment attached to the scale but not the spectra of the surrounding shale (Fig 3). Finally, there were small peaks at m/z 485.10 and 499.10 in the negative spectra of the fish fossil that can be assigned to porphyrins and that map to the same areas as the porphyrin signals in the positive ion spectra (S1 Table). These peaks were not detected in the spectra of any the analyzed sediment and shale samples.

In addition to the peaks that can be assigned to porphyrins, peaks that can be assigned to fragments of polycyclic aromatic hydrocarbons (PAHs; m/z 77.04, 91.05, 115.05, 128.05, 139.05, 141.06 etc.; Figs 2, 3 and S2–S5) were observed in the spectra from the fossil, the reddish white-brown sediment and the nearby collected shale sample [15, 31]. The porphyrin peaks, especially for peaks below m/z 435, were somewhat obscured by the repeating pattern of peaks originating from PAHs (Greenwalt, Rose et al. 2015) as certain PAHs produce peaks with similar masses as porphyrins. Contrary to porphyrins, peaks attributable to PAHs showed an overall steady decrease in intensity with increasing mass as observed in for example the spectrum of the shale (Fig 2D) [15]. No peaks that can be assigned to microbial biomarkers such as hopanes were observed in any of the analyses [32–34].

**Fig 4:**
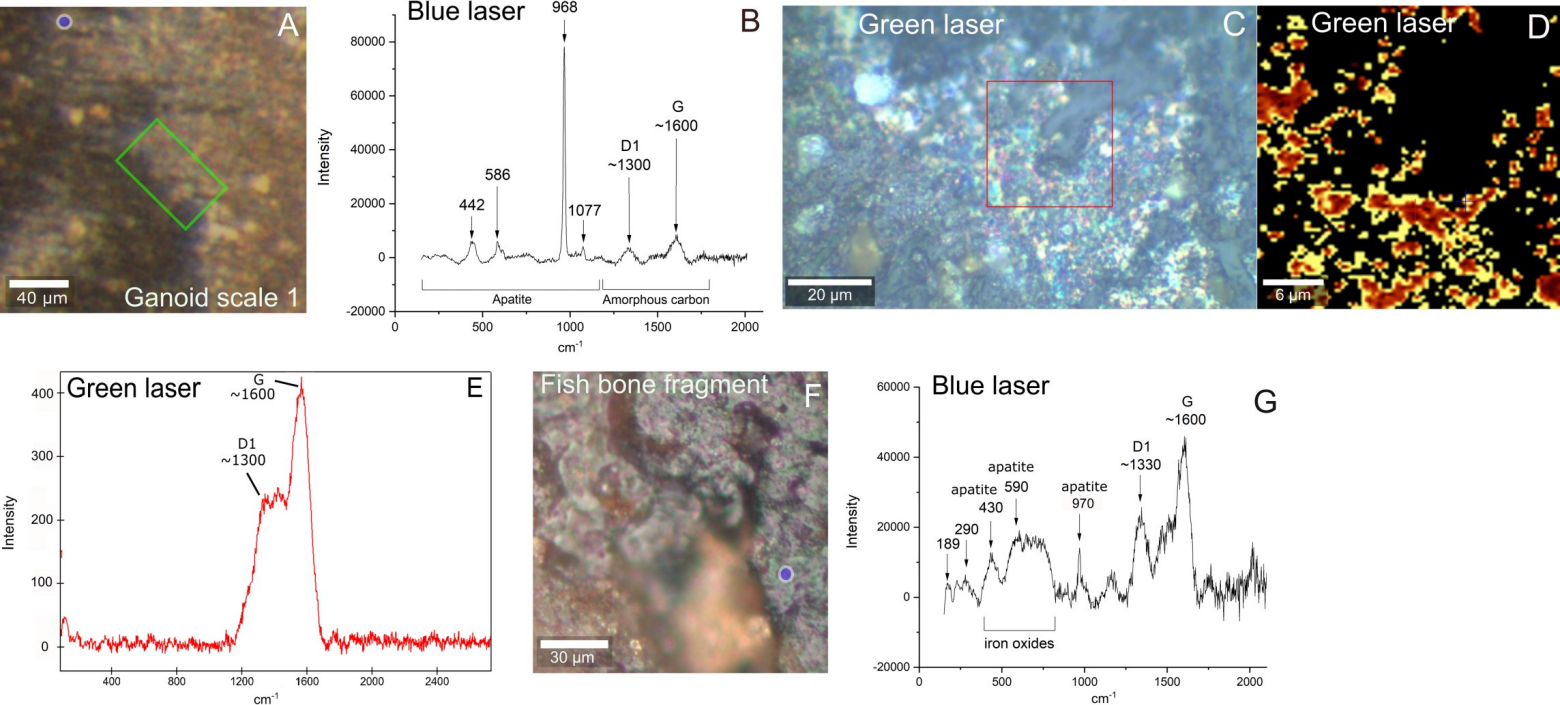
Chemical and mineralogical characterization of Messel fossil scale and bone fragment by Raman. A) Micrograph of ToF-SIMS area on ganoid scale 1 with gray-brown film (Fig 1A and 1B). B) Blue laser Raman spectrum from blue circle in area of film on fish scale in A. C) Micrograph of area in green square in A where green laser Raman scan was performed. Note that scale is rotated compared with A. D) Raman map of D1 peak of red area in C. E) Summed Raman spectrum of scan in red square in C) indicating refractory carbon. F) Micrograph of ToF-SIMS area on bone fragment (Fig 1E and 1F). Blue circle indicates area of Raman analysis on porphyrin-rich area identified by ToF-SIMS. G) Blue laser Raman spectrum of blue circle on bone fragment in F.

Raman spectroscopy, either using a blue and green laser, of the same area as the one of ToF-SIMS analysis show, in addition to high background fluorescence, clear peaks at ⁓1350 and ⁓1600 cm^-1^ which are characteristic of the D1 and G peaks that are commonly seen in refractory organic materials (Fig 4) [35].

### Reflected light microscopy and SEM of fossil, sediment, and shale

**Fig 5:**
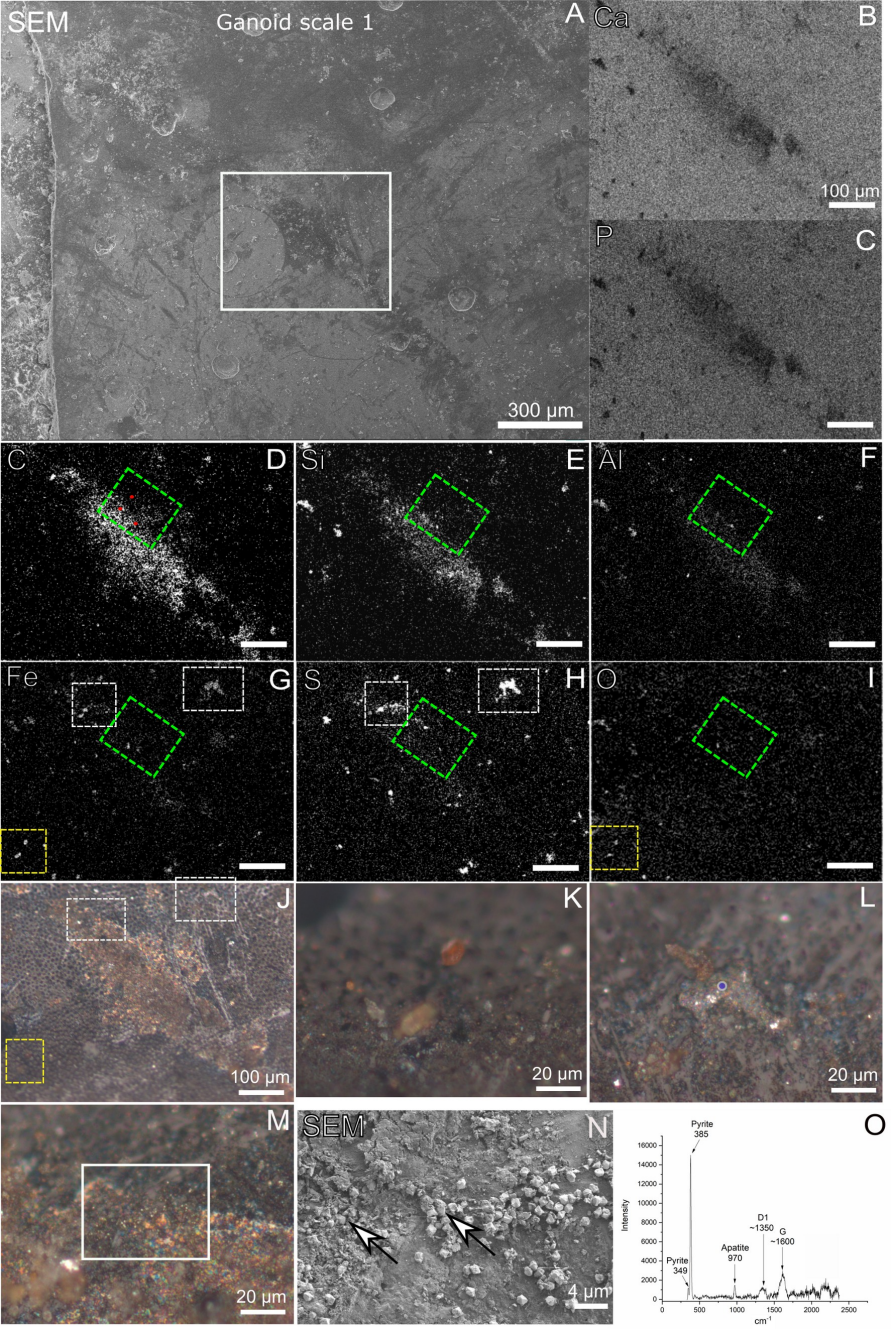
Chemical and mineralogical characterization of gray-brown film on Messel fossil scale by EDX and Raman. A) SEM of gray-brown film on ganoid scale 1. Note that the ganoid scale is rotated compared with Figs 1, 2 and 4A. B-I) EDX maps of white square in A. Green square in D-I show area of ToF-SIMS analysis. Red dots in D indicate area of point spectra in S2 Table. J-M) Micrographs of ganoid scale 1. Yellow square in J indicates area of overlapping Fe and O signals and of micrograph in K. White squares in J indicate areas of Fe and S overlapping signals and of micrographs in L and M. N) SEM image of white square in M. White arrows show pyrite crystals intermixed with gray-brown film. O) Blue laser Raman spectrum obtained from blue circle in L, showing presence of pyrite in highly reflective phase infilling pore in fossil.

**Fig 6:**
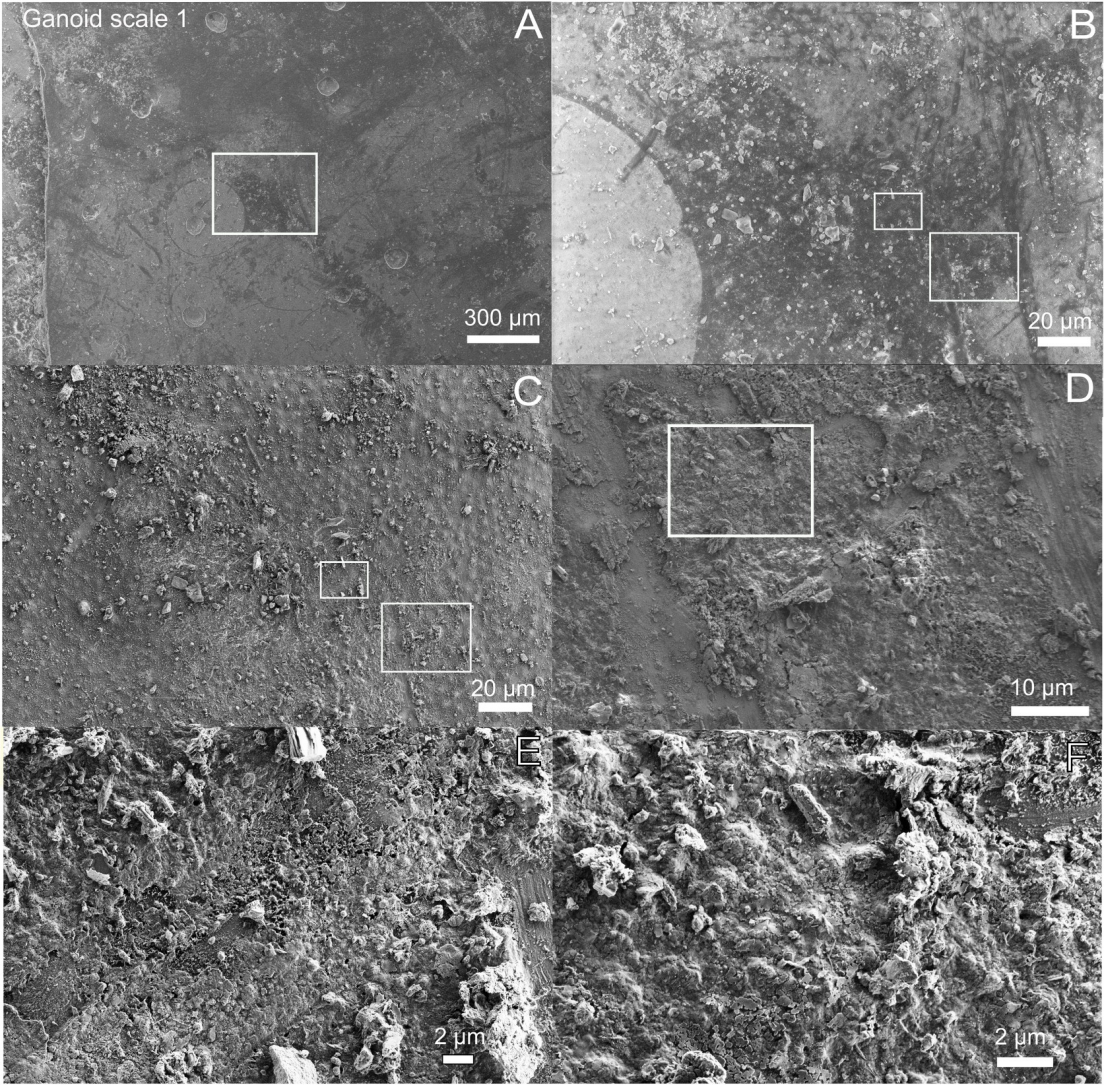
Ultrastructure of gray-brown film on Messel fossil fish scale. A) Low-vacuum SEM image showing gray-brown film on ganoid scale 1. The film appears darker than the rest of scale. Note that the ganoid scale is rotated compared with Figs 1, 2 and 4A. B) Zoom-in of white square in A. C) Same area as in B but in high-vacuum mode. D) Zoom-in of right white square in C. E) Zoom-in of left white square in C. F) Zoom-in of white square in D.

Reflected light microscopy of the gray-brown film on scale 1 shown in Fig 1A and 1B revealed the presence of a shiny red-brown (even iridescent) phase (Fig 5). In SEM images the film appeared desiccated and crisscrossed with cracks (Fig 6). Mineral grains were observed to be embedded both within the film and lying loosely on top of the scales and bones (Figs 5J–5M, 6 and S1 and S6). A small number of microbodies (600-700nm) were present in one area of the analyzed bone where porphyrin peaks were found (S4J Fig). SEM of the bone area with where the porphyrins were detected indicated that, especially the non-bony structure, were quite porous and fibrous (S4K and S4L Fig).

Both light microscopy and SEM imaging revealed that the nearby collected shale sample was contaminated by fungal hyphae and associated spore bodies (S1K and S1L Fig), which was not the case for the fossil and the sediment attached to the fossil.

### Inorganic signals from fossil, sediment, and shale

When both organic and inorganic (Al^+^, Si^+^, Mg^+^ and Al_x_Si_y_O_z_^-^ clusters) ion signals were mapped from the ToF-SIMS analyses, an interesting pattern emerged (Figs 2I–2S and S2, S4 and S5). The ion images of the scales and bones of the fish showed that signals of inorganic ions such as Al^+^, Si^+^, Mg^+^, AlSiO_4_^-^ and AlSi_2_O_6_^-^ co-localized with the signals from the organic material (Figs 2I–2S and S2, S4 and S5) in most areas. However, in the area of the gray-brown film on the fish scale, the signals of Al^+^, Si^+^, Mg^+^, AlSiO_4_^-^ and AlSi_2_O_6_^-^ instead decreased. The obtained EDX maps of the scale with gray-brown film showed a good correlation with ToF-SIMS results with an increase in C signal and decrease of Ca and P signals in the area of gray-brown film (Figs 2I–2S and 5A–5I and S2 and S2 Table). However, the EDX analyses also showed an increase of Al and Si signals in this area, something that was not observed in ToF-SIMS maps of Al and Si (Figs 2, 5 and S2). The difference in spatial distribution of Al and Si in ToF-SIMS and EDX can be explained by the deeper interaction volume of EDX (up to several μm depending on the element) compared to ToF-SIMS (a few nm) indicating that Al and Si were probably located beneath the carbon/organic layer [36]. However, specific identification of the mineral phase containing the Al and Si signal was not possible though it is highly likely an aluminosilicate, especially considering presence of Al_x_Si_y_O_z_ clusters in the ToF-SIMS data (Figs 2I–2S and S2 and S5 and S1 Table).

The EDX maps of the fossil also showed the presence of iron, which was either co-localized with sulfur, carbon and/or oxygen (Figs 5 and S6 and S8). Reflected light microscopy of the areas on the scale where iron co-localized with sulfur revealed the presence of microcrystals with a high reflective index which infilled pores in the fossil or were trapped in the gray-brown film (Fig 5L and 5M). Raman spectroscopy of the microcrystals in the pores (Fig 5L) showed they consisted of pyrite (Fig 6O). SEM of the microcrystals trapped in gray-brown film showed the presence of subeuhedral grains (Fig 5N), also consistent with pyrite.

Reflected light microscopy of the areas where the iron co-localized with carbon and/or oxygen, showed mineral grains which were not very well attached to the fossil (Figs 5K and S6K and S6L). Raman spectroscopy of the iron and carbon rich grains revealed that they consisted of mainly of siderite (S6M Fig), while Raman spectroscopy of grains consisting of iron and oxygen (S6K Fig) only showed fluorescence and were easily transformed by the laser beam. We interpreted these iron and oxygen phases as poorly crystalline iron oxides, possibly formed due to weathering of other iron phases such as pyrite and siderite in the sample. Peaks that can be tentatively assigned to iron oxides are also observed in Raman spectra of the sediment attached to the fossil (S6M and S7 Figs). These observations were corroborated by the EDX analyses, that showed areas were sulfur co-localized with calcium and oxygen, potentially indicating the presence of a calcium sulfate phase (not confirmed by Raman and XRD) (Figs 5 and S6).

XRD and Raman of reddish white-brown sediment, which formed a crust on the studied subsamples of the fossils (Figs 1 and S1, S6A and S6L and S8), identified siderite, iron oxides, pyrite and tentatively small amounts of clay (S6M and S7A–S7C Figs). This is consistent with elemental data from EDX (S2 Table and S8 Fig) and inorganic data from ToF-SIMS (S7D Fig), which also identified a poorly crystalline calcium phosphate phase in the sediment. Microscopy and EDX of a cross section of the vertebra/sediment interface showed a pyrite-rich zone closest to the fossil followed by a layer of siderite and calcium phosphates (S8 Fig). These results are consistent with literature reporting that many fossils in Messel are encrusted with siderite and calcium phosphates [37, 38].

The ToF-SIMS, EDX and XRD analysis of the shale sample collected nearby the fossil showed it is dominated by silicates such as clays and quartz with minor amount of siderite and pyrite present (S2 Table and S7 Fig).

## Discussion

The ToF-SIMS peak patterns shown in Figs 2, 3 and S3 have been shown to be typical of porphyrins, including heme [13, 16, 29, 30]. The porphyrin peak pattern of the fossil more closely resembles the one observed in the spectra of the heme b standard and diagenetic altered heme in the abdomen of the fossil mosquito than the one in the spectrum of the chlorophyll *a* standard (Fig 3A–3H) [13, 16].

Different porphyrin derivatives have previously been detected in the Messel oil shale by hydrous pyrolysis or extraction followed gas chromatography mass spectrometry [7, 8, 39–42]. The precursors of these have been identified as chlorophylls, bacteriochlorophylls and heme [8, 39]. Therefore, the porphyrin peak pattern observed in the fish fossil (Figs 2, 3 and S3) could originate from extraneous inputs such as the sediments where the fish settled after death. However, the mass spectral peak distribution of chlorophylls and bacteriochlorophylls in their native form differ significantly from the one of the heme b standard and the one detected in the fossils (Fig 3) [16, 30]. Additionally, theoretical mass calculations of the porphyrin derivates (e.g. etioporphyrins and deoxophylloerythroetioporphyrin) identified in the Messel shale [39, 40], suggest different peak distribution patterns for these porphyrins, both in terms of exact molecular mass and their gaussian distribution pattern, compared with the pattern observed in the fish fossil spectra (Figs 2, 3 and S3). This can be attributed to vinyl reduction of side chains, and the exchange of Ni^2+^ for the central Mg^2+^ (if chlorophyll) in these compounds. The vinyl reduction reaction makes the porphyrin derivates in the Messel shale more saturated than intact heme or slightly diagenetically altered heme as detected in fish and mosquito fossils (Fig 3) [39, 40]. The added H and different co-factors (as in chlorophyll) shift the mass of the peaks upward both relative to the nominal mass and the actual mass (the mass difference ^24^Mg versus ^58^Ni is m/z 33.95) [29]. Analyses did identify similar but not identical porphyrin peak pattern as the one detected on the fish fossil, in the sediment attached to fossils but not in the shale. However, the signal was only detected in 1 of 6 areas analyzed and as the sediment was attached to the fossil this signal could originate from the fossil, though an exogenous source cannot be completely ruled out but is considered unlikely. On the fish fossil the porphyrin pattern was only found in specific areas tied to specific morphological features. If the porphyrin signal on the fossils was exogenous in origin it would be expected more homogenously distributed across the fossil than what was observed.

Therefore, it is more likely that the porphyrin signal on the fish fossil either originates from heme sourced from blood or cytochromes in the fish. A further possibility is that microbes, that colonized the fossils after death may have contributed to the heme signal, however, only one of the three areas where heme was detected in the fossil revealed the presence of microbodies, which could be either melanosomes or microbial fossils (S4J Fig). Therefore, we discount them from contributing to the detected heme signal [10, 43, 44]. Additionally, bacterial biomarkers such as hopanes were not detected in this study [32, 34] and anaerobic organisms, the most likely organisms to colonize the carcass, express very low levels of porphyrin containing cytochromes [45]. The areas where heme was localized on the fossil, such as near the Williamson canals, on a scale where the outer ganoid layer have broken of exposing the laminar bone (where blood vessels are located) and on a porous piece of bone, are all consistent with where blood would be found in a decaying carcass. Another clue to the origin of the heme signal in the fish fossil is its localization to the top layer of the gray-brown film as revealed by the comparison of ToF-SIMS and EDX data (Figs 2 and 5). This can be interpreted as blood from the fish mixing with sediment postmortem with the sediment then settling below the blood. Combined these observations are best explained by blood/cytochromes of the fish being the source of the heme signal in the fish fossils though contribution from a microbial source cannot completely be ruled out.

Although there is a close match of the detected porphyrin peak pattern to the heme standard, the porphyrins in the fish fossil have been diagenetically altered to some degree (Figs 2, 3 and S3). Similarly to the spectrum of diagenetically altered heme in a fossilized mosquito from the Kishenehn formation [13], the spectra of the fish fossil lack the molecular ion of the heme at m/z 616 and its fragment ion at m/z 557. Furthermore, the highest repeating set of peaks centered around m/z 495 and 497 show a decrease in intensity compared to the heme standard probably due to decarboxylation and dealkylation of the native heme [13]. However, further comparisons with the heme spectrum of the fossilized mosquito [13] does reveal additional alteration of the spectra of the fish fossils (Fig 3). The most obvious difference is the relatively strong peaks at nominal masses m/z 457, 471, 485 that are 2 u above the central peaks of the intact heme (e.g. m/z, 455, 469, 483) (Fig 3 and Table 1). The difference in the porphyrin pattern could be explained by exchange of central iron with nickel and/or vinyl reduction of the side chains. These changes are known to shift the main central mass of the gaussian distribution about two and/or four masses higher [29]. The nickel interpretation is corroborated the localization of Ni^+^ and NiC_2_N_2_^-^ to the same areas as the peaks assigned to heme (Figs 2, 3 and S4–S5) [46]. Though the peaks assigned to these compounds are also observed in spectra of the sediment attached to fossil, their relative intensities are significantly lower than in the fish fossil (Table 2).

**Table 1:** Ratios of porphyrin peaks.

Peak ratio	Heme b	Mosquito	Scale 1
457/455	0.76	0.69	0.95
471/469	0.79	0.65	0.83
485/483	0.62	0.58	0.68

**Table 2:** Ratios of peaks assigned to nickel and organically bound nickel.

Sample	Ni (divided by total ion)	NiC_2_N_2_/FeC_2_N_2_
Scale 1	0.00080	0.84
Reddish white-brown sediment	0.00040	0.27
Mosquito	0.00007	0.02

This is first time this known metal exchange mechanism (Ni for Fe) in porphyrin diagenesis has been spatially correlated and therefore directly observed within a vertebrate fossil. Interestingly, it can be inferred from the peak pattern of the detected heme in the fish fossils that the exchange of the central metal ion of the heme appears to have happened early in diagenetic transformation of the porphyrins, before for example the vinyl reduction of the side chains, possibly due to access to abundant Ni. Previously it has been proposed that the complexing with the metal ion is the final step in the transformation of porphyrins to geopophyrins, as this reaction requires the highest temperatures [47, 48]. However, studies of porphyrins in the Messel oil shale have previously shown anomalous preservation pathways such as complexation with metal ions before decarboxylation and dealkylation, though not before vinyl reduction [48]. Also it known that Messel shale itself has not been exposed to temperatures much above 40°C [8], which suggest that the diagenetic transformation of porphyrins in these oil shale systems can take numerous pathways and does not necessarily rely on high-temperature reactions [39, 42].

Raman spectroscopy identified the presence of refractory carbon in the gray-brown film on the scale that contained heme (Fig 4). However, individual bands of porphyrins or for that the matter any other organic molecules were difficult to distinguish in the collected spectra [49, 50]. This could be due to the high background fluorescence, lower sensitivity of Raman compared to ToF-SIMS or possibly laser-induced breakdown of the heme [49]. It appears most likely that the higher sensitivity of the ToF-SIMS instrument (ppb) compared to Raman spectroscopy (wt%) is key in the detection of specific organic species within complex refractory organic material. A previous study of fossils with Raman have claimed to be able been to detect porphyrins in fossils [51]. However, these studies were recently been questioned and it was suggested that the signals observed the in the Raman spectra are background subtraction artefacts from the spectral background of the instrument [52].

The origin of the detected PAH:s is less clear than the porphyrins, as they are found in spectra of the sediment attached to the fossil and of the surrounding shale (Figs 2 and S2, S4 and S5). This is not surprising as previous ToF-SIMS analyses have shown that spectra of shales are normally dominated by peaks that can be assigned to PAHs [31]. The spatial distribution of the PAHs indicates they have mixed origin, i.e., from the fish fossil (Fig 2) and dissolved or particulate organic material deposited along with the sediments.

The ToF-SIMS and EDX analysis indicate strong association between the organic signals, such as porphyrins and PAHs, and inorganic signals such as silicon, aluminum and Si_x_Al_y_O_z_ clusters (Figs 2, 5 and S2, S4 and S5). There are of siliceous algal fossils in Messel oil shale [53, 54], however, no algae or diatom structures were identified in the fossils and the co-localization of Si and Al and the SEM images suggests the presence of aluminosilicates such as clays. Indeed, clays such as smectites (nontronite), illite and kaolinite have previously been identified in the Messel shale [37, 54, 55]. Unfortunately, there is not enough material to obtain XRD data from the fossil itself but the XRD of the shale sample collected near to the fossil and of reddish white-brown sediment attached to the fossil, indicate the presence of a clay (S7B and S7C Fig). Lack of strong signals of magnesium, sodium and calcium coupled with a silica to aluminum ratio that varies between 1.3–2.1 (from EDX analysis of the fossils), are consistent with the presence of either kaolinite or smectite clays (S2 Table). The exact origin of the clay in the gray-brown film (Fig 1) and whether it is authigenic or not is unknown. However, regardless of origin, the presence of clay such as a kaolinite would to some extent promote the preservation of the organic material [56–61]. In many exceptionally preserved fossils clays often appear as thin sheets covering the organic matter in the fossil, which is similar to how the aluminosilicates here form a layer underneath the organic one in the gray-brown film on the scale [62].

The variation in complex mineral assemblages present in the fossil (aluminosilicates, carbonates, pyrite, iron oxides and calcium phosphates) and associated organics are commonly seen in the exceptional preserved lagerstatten fossils and can be explained by varying pH across the fossil [59, 63, 64]. In this study the organic matter in the fossil, such as porphyrins, seems to be associated with clays while siderite and calcium phosphates are enriched in the reddish white-brown sediments that formed a crust around the fossil compared with the shale sample not in direct contact with the fossil (Figs 1, 2, 5 and S1, S2 and S4–S8). The following scenario could possibly explain the presence of heme on the fish and the associated minerals. The expired fish was deposited into the fine-grained agrillaceous sediments of the Messel lake [2]. The euxinic to semi-euxinic conditions of the sediments limited aerobic degradation of the carcass and promoted microbial sulfate reduction by sulfate reducing bacteria (SRB). H_2_S released in this process combined with Fe in the sediment to produce pyrite which filled some cavities of the fossil and covered some fossil surfaces (Figs 5L and S8) [2, 63]. At an early point in diagenesis, possibly during settling or premortem predation of the carcass some scales were damaged, and blood was released from the fossil, possibly mixing with the surrounding sediments which included clays (Figs 2, 5 and S2, S4 and S5). The ongoing sulfate reduction degraded some of the organic matter, however, the iron and the aluminum present, as well as the clay itself protected remaining organics including heme from being completely degraded [56–61]. The presence of siderite attached to the fossils (Figs 1 and S1, S6–S8 and S2 Table) indicates that sulfate was depleted at some point during diagenesis, which enabled precipitation of iron as siderite by methanogenesis [65, 66]. Methanogenesis as the origin of the siderite in Messel is supported by isotope studies that showed positive δ^13^C values in the siderite, including the siderite attached to fossils, and the presence biomarkers specific for methanogenesis in Messel sediments [8, 37, 38, 67]. The enrichment of the calcium phosphate near the fossil suggest it formed from P released from the degradation of organic material in the fossil and/or from the diagenetic alteration of bones and scales (S7 and S8 Figs and S2 Table) [37, 63, 64, 68–70]. Eventually the bacterial activity completely ceased around the fossil possibly due to the crust of minerals (S1 and S8 Figs) which had formed around the fossil and which restricted access to the fossil from the surrounding sediments and therefore minimized further microbial colonization and degradation [71]. The formation of siderite encrustation around fossils by methanogenesis is not uncommon in fossils [72, 73] and exceptional preservation of fossils and organic material are often reported from carbonate (including siderite) concretions [71, 73–76]. Interestingly stratigraphic correlation between siderite layers and fossil abundances have been reported for other maar lakes though such studies have so far been inconclusive for Messel [77]. It is clear that to fully understand the exceptional preservation of fossils in Messel more extensive studies are needed.

This is the third instance that heme derivates have been detected in fossils, the other being in the engorged abdomen in a female mosquito from the Middle Eocene Kishenehn Formation, Montana [13], and in the skin of turtle from the Eocene Fur Formation, Denmark [46]. In addition, heme has been tentatively detected inside Cretaceous dinosaur bone [78]. Messel and Kishenehn are both lacustrine settings while Fur is a marine setting with volcanic input [13, 46]. In several of these sites the fossils are to some extent associated with clay and carbonates. For example, the Fur turtle was found in a carbonate concretion and the preserved heme is in this fossil is associated with clay minerals [46]. The fossilized mosquito from the Kishenehn Formation with preserved heme was found in a carbonate rich stratigraphic layer, covered by a thin layer of aluminosilicates [13, 31]. In fact this aluminosilicate layer had to be removed in order to expose the carbon rich fossils for analysis [13]. This combination of carbonates and aluminosilicates are similar to what we have observed in the Messel fossil reported here. Additionally all settings are considered to have reached low thermal maturity which is important for the preservation of organic molecules [8, 46, 79]. The detection of heme in all three localities indicate that the preservation of heme in fossils is not rare and probably extends beyond the Eocene.

The metal-binding capability of porphyrins has important implications for the interpretation of metal signals and preservation of organic molecules and soft tissues in fossils even if organic molecules are exogenous. For example, the distribution of elemental signal, especially ones associated with purported melanosomes, in fossils have been proposed as a way to identify organs in fossil soft tissue of vertebras [80]. However, as it has been shown that porphyrins may be preserved in fossils together with melanosomes this would complicate the interpretation of these signals, especially as porphyrins are known bind to a variety of metal ions [46, 48]. Additionally, heme might have an important role in stabilizing soft tissue as a provider of reactive iron to initiate cross-linking processes [81].

## Conclusion

We identified porphyrins most likely heme, on scales and bones from a fish fossil from the Messel pit which is most likely sourced from the blood/ cytochromes of the fish itself though a microbial contribution cannot be completely ruled out. The detection in this fossil and in fossils from other locations indicates that heme derivates and other porphyrins are common in the fossil record. Associated with the porphyrins and other organic molecules are aluminosilicates, pyrite, siderite, calcium phosphates and iron oxides. The close association of both aluminosilicates and carbonates appears to be important for the preservation of heme and possibly other organic molecules and soft tissues in the fossil record. This study showed that heme has been diagenetically altered by exchange of metal ion from iron to nickel, which is first time metal exchange in a porphyrin has been spatially correlated to a vertebrate fossil. Porphyrins are important biomolecules and understanding their preservation and diagenesis in the fossil record is important. This study has shown it is possible to detect and potentially differentiate between porphyrin on a submicron spatially resolved level and therefore shed valuable light on the diagenetic processes in fossil lagerstatten.

## Supporting information

S1 Fig Micrographs and SEM images of Messel fossils, sediments, and shale. A) Ganoid scales as received. B) Bone fragments as received. C) Vertebra including cut part showing cross section of vertebra and sediment. Red box indicates area of zoom-in of the cross section. Black dotted box indicates area of S8A Fig. D) Backside (base) of ganoid scale 1 with reddish white-brown sediment attached. E) Reddish white-brown sediment removed from ganoid scale 1 in D and attached to Si wafer with tape. F-H) Ganoid scales and bone fragments where no porphyrins were detected. Red arrows in F-G indicate apertures in ganoid layer of fish scales. I) Piece of nearby collected Messel shale. J) Zoom-in of piece of Messel shale showing snail fossil in white box in I. K) Zoom in of hyphae of fungi in green box in I. L) SEM image of hyphae and spores of fungi.
(TIF)

S2 Fig Extended ToF-SIMS data of gray-brown film on Messel fossil fish ganoid scale 1. A) Micrograph of ToF-SIMS analysis area on ganoid scale 1. High spatial resolution ToF-SIMS ion images (500x500μm^2^) of B) sum of porphyrin peaks (*m/z* 441, 455, 469 and 483), C) sum of PAHs (*m/z* 77, 91, 139, 141 and 165), D) Al^+^, E) Si^+^, F) Mg^+^ and G) sum of AlSiO_4_^-^ and AlSi_2_O_6_^-^.
(PDF)

S3 Fig ToF-SIMS spectra of ganoid scales and bone fragments of Messel fossil fish. Positive ToF-SIMS spectra (m/z 410–530) of A) area indicated by green dotted box on ganoid scale 1 with gray-brown film in Fig 1B, B) area indicated by green dotted box on fish bone fragment in Fig 1F, and C) area indicated by green dotted box in ganoid scale 2 in Fig 1D. D-F) Zoom-in of green dotted box in spectra in A-C showing *m/z* 448–461.
(PDF)

S4 Fig ToF-SIMS ion and SEM images of Messel fish fossil bone fragment (Fig 1E and 1F). A) Micrograph of ToF-SIMS analysis area on bone fragment. High spatial resolution ToF-SIMS ion images of B) sum of porphyrin peaks (*m/z* 441, 455, 469 and 483), C) Fe^+^, D) Ni^+^, E) sum of PAHs (*m/z* 77,91, 139, 141, 165), F) Al^+^, and G) Si^+^. H) SEM image of same area as in A. I) Zoom-in of area indicated by right white box in H where porphyrins were detected. J) Zoom-in of area indicated by white box in I with microbodies (white arrows). K) Zoom-in of area indicated by left white box in H with whitish non-bony structure (Fig 1E and 1F). L) Zoom-in of area indicated by white box in K showing fibrous structure (white arrow).
(PDF)

S5 Fig ToF-SIMS ion images of Messel fish fossil ganoid scale 2 (Fig 1C and 1D). A) Micrograph of ToF-SIMS analysis area on ganoid scale 2. High spatial resolution ToF-SIMS ion images of B) sum of porphyrin peaks (m/z 441, 455, 469 and 483) C) Fe^+^, D) FeC_2_N_2_^-^, E) Ni^+^, F) NiC_2_N_2_^-^, G) sum of PAHs (m/z 77, 91, 139, 141, 165), H) Al^+^, I) Si^+^, K) Mg^+^ and K) sum of AlSiO_4_^-^ and AlSi_2_O_6_^-^.
(PDF)

S6 Fig SEM/EDX images of Messel fish fossil bone fragment (Fig 1E and 1F). A) Micrograph of area of ToF-SIMS analyses on fish bone fragment. B) SEM image of area indicated by white dotted box in A. Green square indicates area of ToF-SIMS analysis in S4 Fig. C-J) EDX maps of area indicated by blue dotted box in B. K) Micrograph of area indicated by white dotted boxes in G and J where Fe and O co-localize. L) Micrograph of area of red dotted boxes in C, G and J showing area where C, Fe and O co-localize. Blue circle shows area of Raman analysis in M. M) Blue laser Raman spectrum of mineral grain attached to fossil showing presence of siderite (1089 cm^-1^), organic material (D1 and G) and iron oxides (⁓400–800 cm^-1^). Peak marked with * can tentatively be assigned to poorly crystalline calcium phosphates.
(TIF)

S7 Fig Raman, XRD and ToF-SIMS of sediments attached to fish fossil and shale collected nearby. A) Blue Raman spectrum of sediment attached to back of ganoid scale 1 showing presence of siderite (1089 cm^-1^), organic material (D1 and G) and iron oxides (⁓400–800 cm^-1^). Peaks marked with * can tentatively be assigned to poorly crystalline calcium phosphates. XRD diffractogram of B) sediment attached to back of ganoid scale 1 and C) shale collected nearby (S1 Fig). D) ToF-SIMS spectra of sediment attached to back of ganoid scale 1 (top) and shale collected nearby (bottom). Notice the difference in mineralogy of sediment directly attached to fossil, mainly siderite, calcium phosphates, iron oxides and pyrite versus shale collected further away which is enriched in silicates.
(PDF)

S8 Fig Microscopy and EDX of fossil/sediment interface of fossil vertebra. A) Micrograph of fossil/sediment interface on fossil vertebra (S1C Fig). Red box indicates zoom-in B). C) Fluorescence micrograph of same area as B. D) EDX maps of same area as A-B showing Ca, P, S, Fe, C and O. The data indicates presence of pyrite, calcium phosphates, iron oxides and siderite in sediment. Note that the area of EDX maps is slightly shifted compared with micrographs in A-B.
(PDF)

S1 Table Ions detected with ToF-SIMS and their assignments. (DOCX)

S2 Table EDX data from Messel fish fossil, sediment attached to fossil and nearby collected shale. (DOCX)
