# Supplementary figures and images for: Detection of porphyrins in vertebrate fossils from the Messel and implications for organic preservation in the fossil record
Source: PLoS One. 2022 Jun 29;17(6):e0269568. doi: 10.1371/journal.pone.0269568 (PMC9242450; doi:10.1371/journal.pone.0269568)

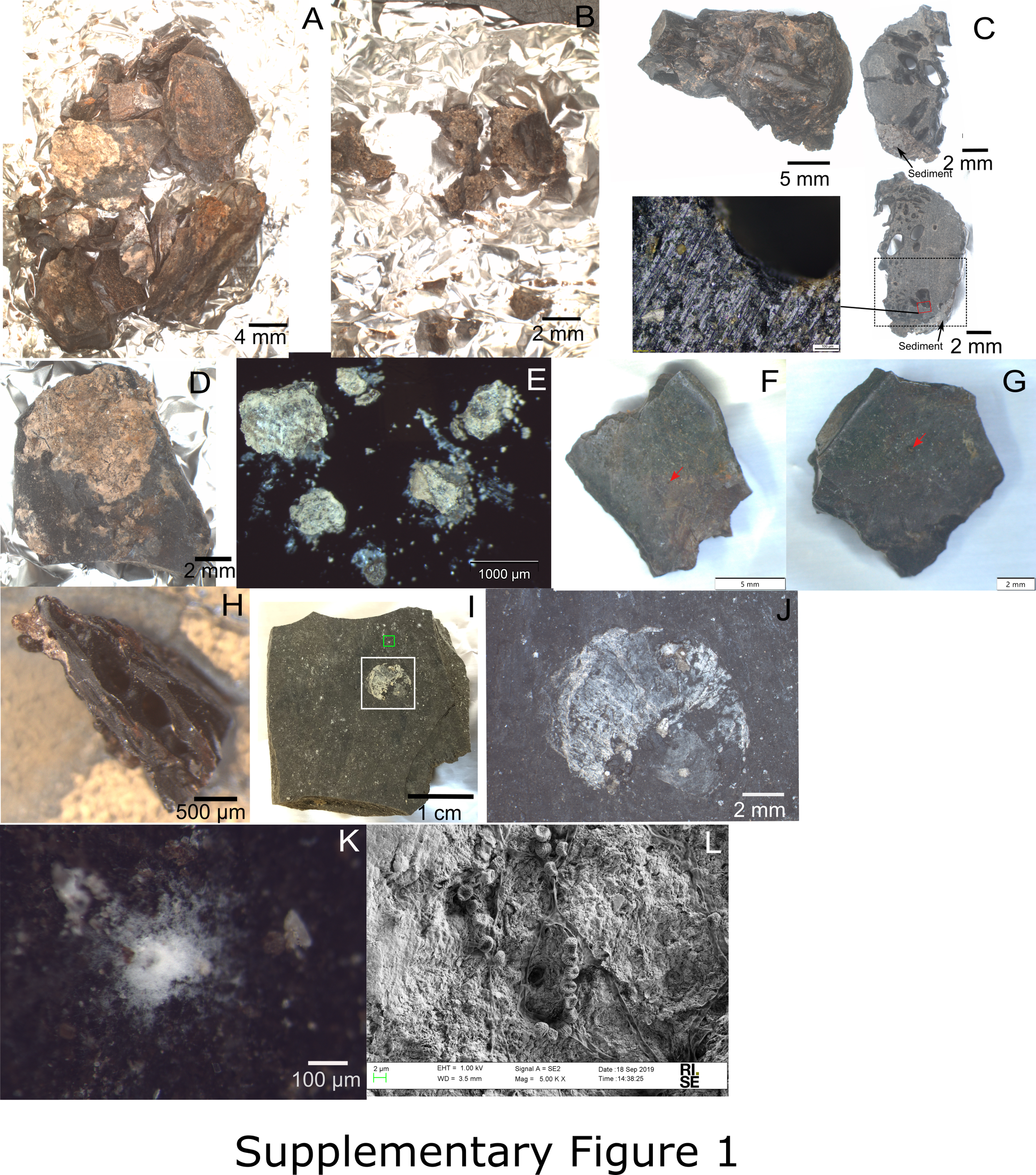

Supplement: S1 Fig — A) Ganoid scales as received. B) Bone fragments as received. C) Vertebra including cut part showing cross section of vertebra and sediment. Red box indicates area of zoom-in of the cross section. Black dotted box indicates area of S8A Fig. D) Backside (base) of ganoid scale 1 with reddish white-brown sediment attached. E) Reddish white-brown sediment removed from ganoid scale 1 in D and attached to Si wafer with tape. F-H) Ganoid scales and bone fragments where no porphyrins were detected. Red arrows in F-G indicate apertures in ganoid layer of fish scales. I) Piece of nearby collected Messel shale. J) Zoom-in of piece of Messel shale showing snail fossil in white box in I. K) Zoom in of hyphae of fungi in green box in I. L) SEM image of hyphae and spores of fungi. (TIF) [file pone.0269568.s001.tif]

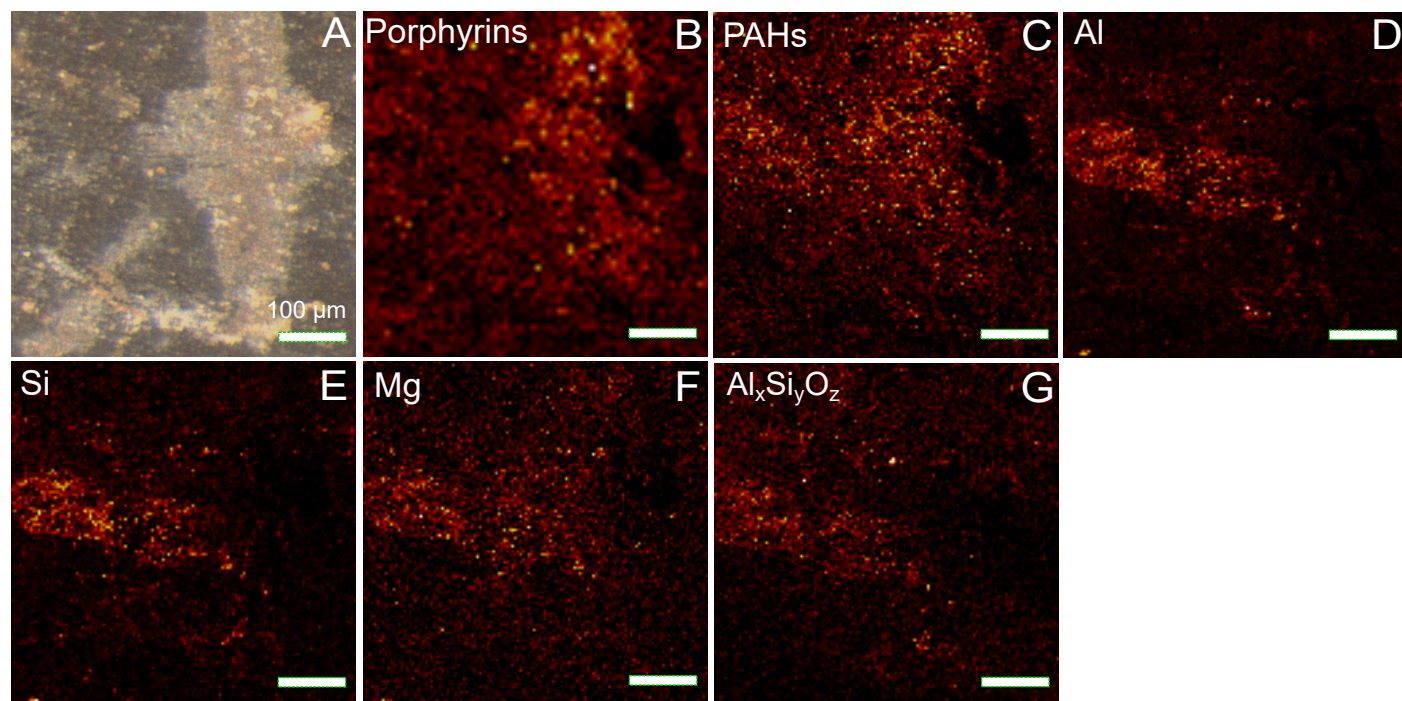

Supplementary Figure 2

Supplement: S2 Fig — A) Micrograph of ToF-SIMS analysis area on ganoid scale 1. High spatial resolution ToF-SIMS ion images (500x500μm2) of B) sum of porphyrin peaks (m/z 441, 455, 469 and 483), C) sum of PAHs (m/z 77, 91, 139, 141 and 165), D) Al+, E) Si+, F) Mg+ and G) sum of AlSiO4- and AlSi2O6-. (PDF) [file pone.0269568.s002.pdf]

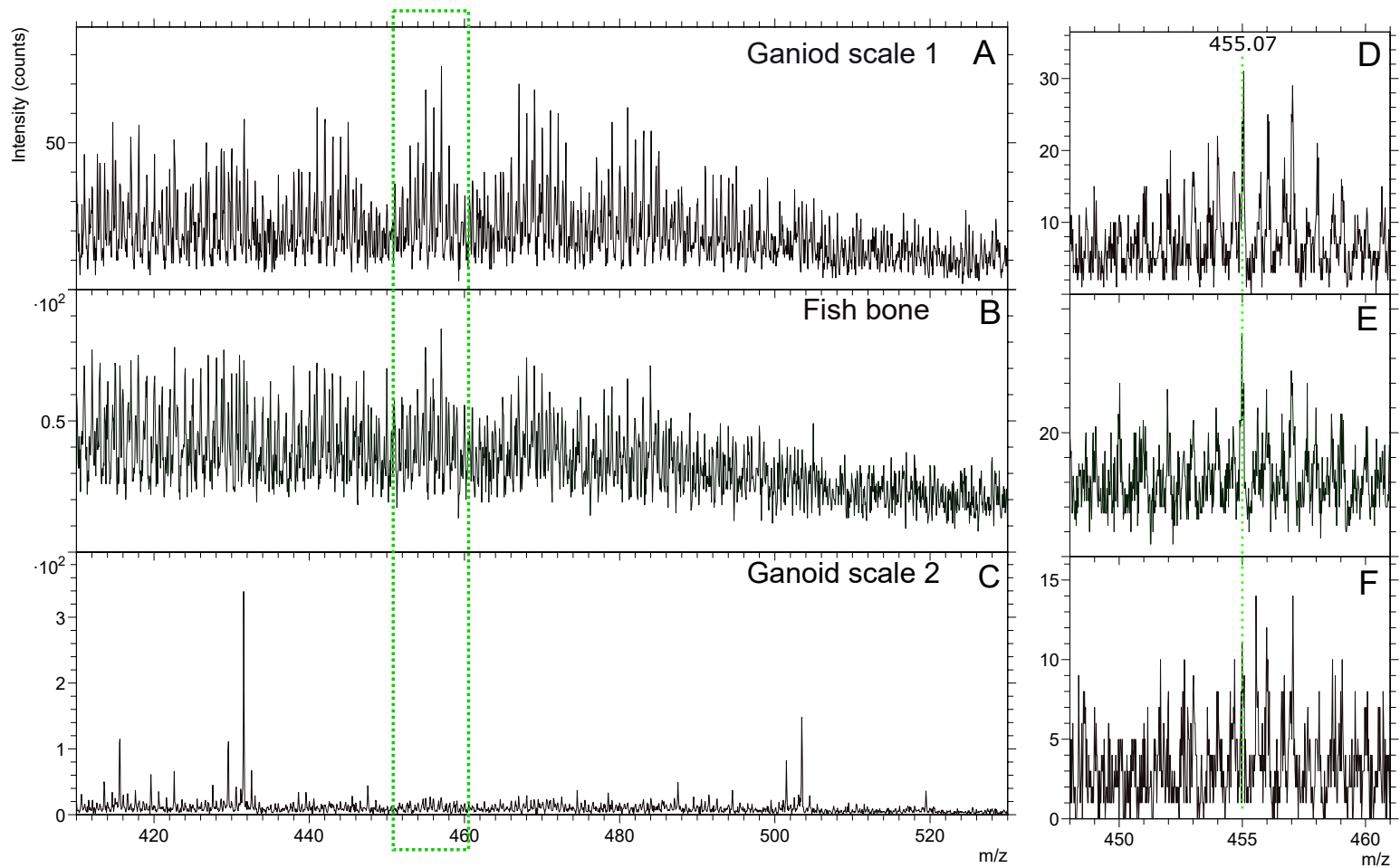

Supplementary Figure 3

Supplement: S3 Fig — Positive ToF-SIMS spectra (m/z 410–530) of A) area indicated by green dotted box on ganoid scale 1 with gray-brown film in Fig 1B, B) area indicated by green dotted box on fish bone fragment in Fig 1F, and C) area indicated by green dotted box in ganoid scale 2 in Fig 1D. D-F) Zoom-in of green dotted box in spectra in A-C showing m/z 448–461. (PDF) [file pone.0269568.s003.pdf]

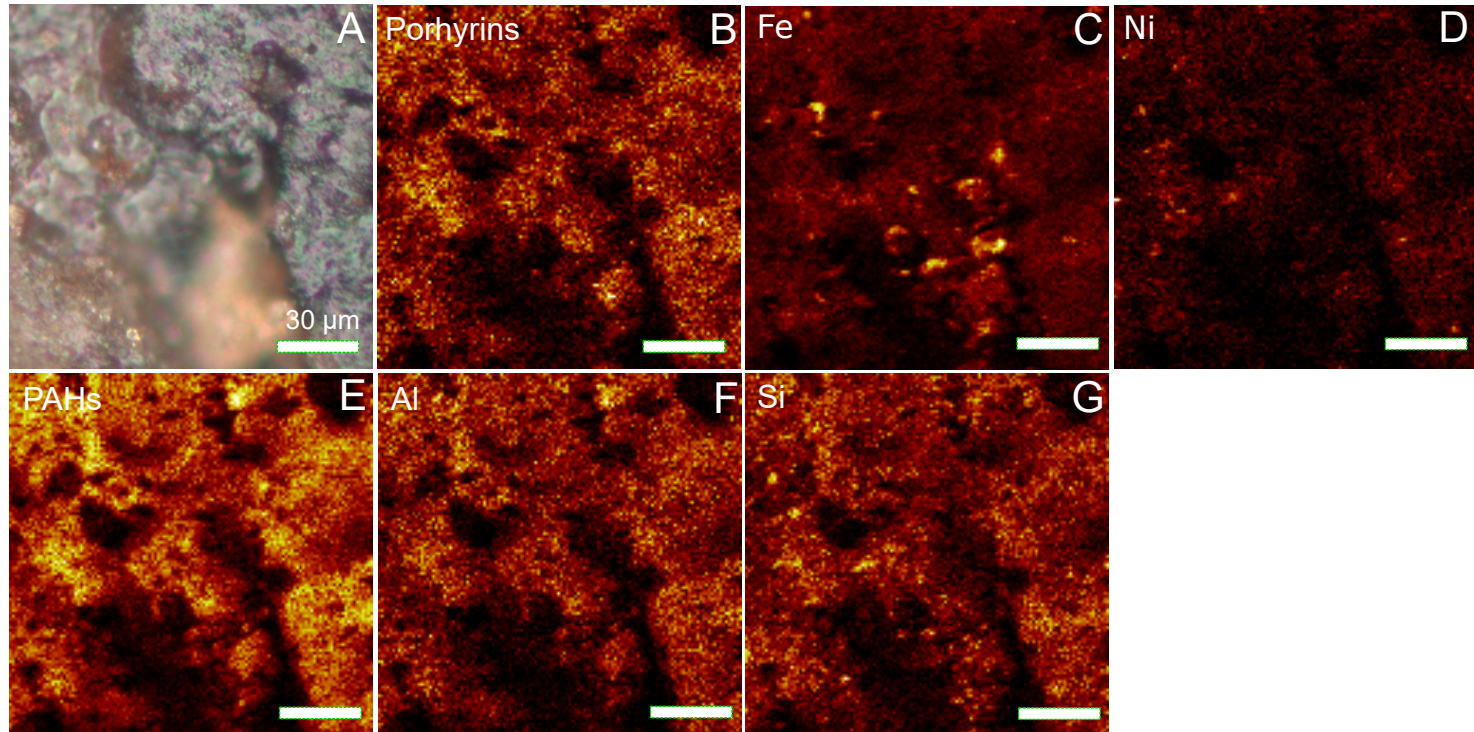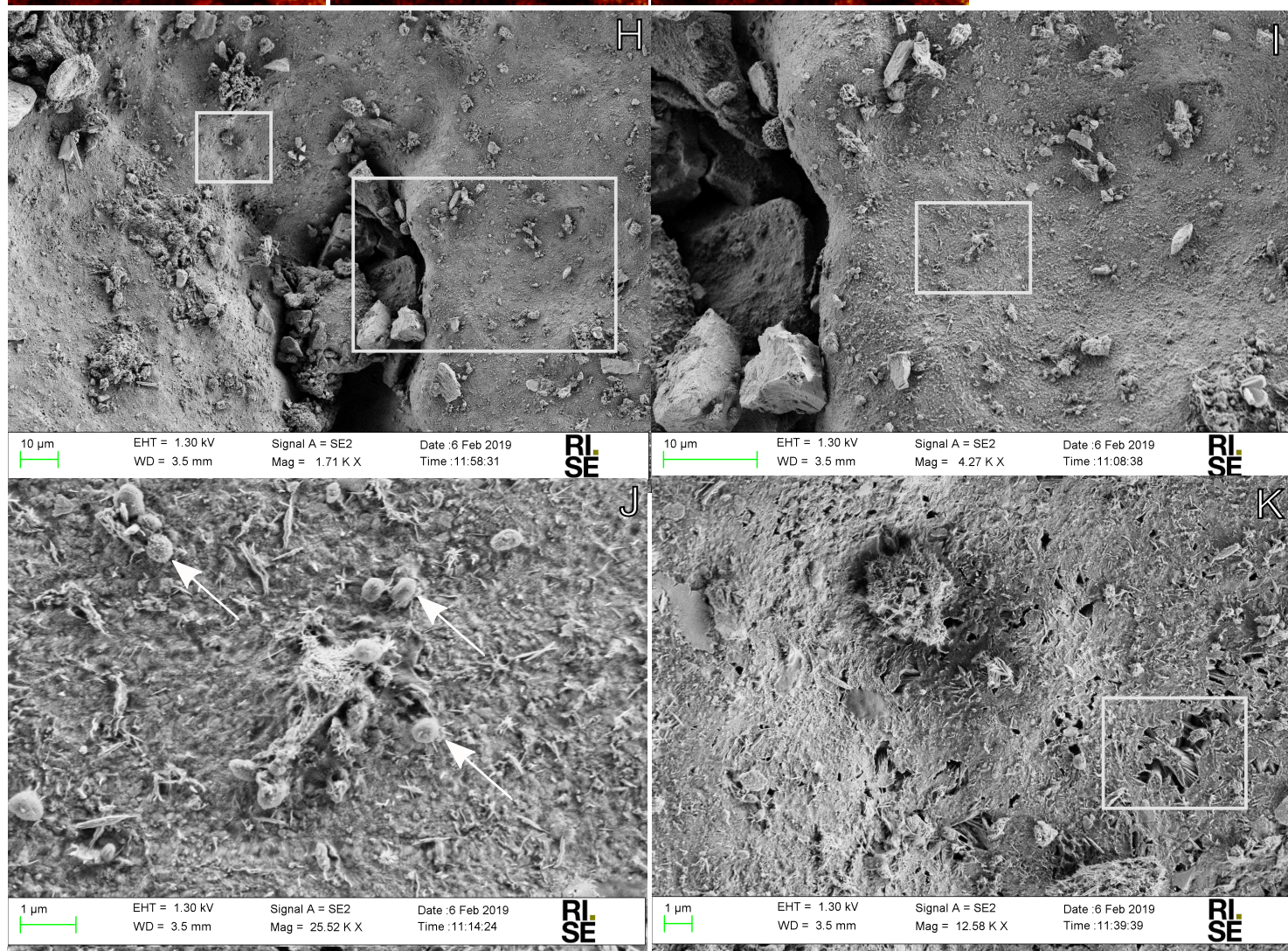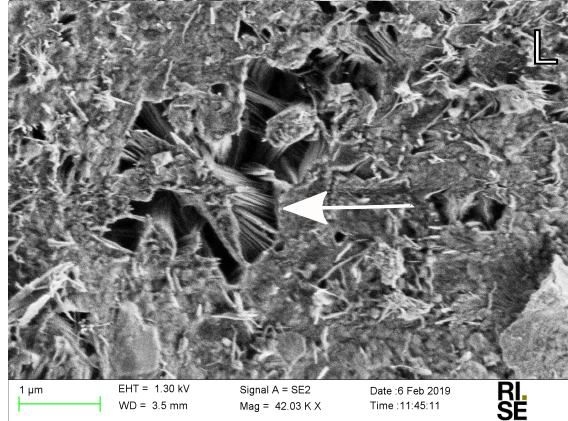

Supplementary Figure 4

Supplement: S4 Fig — A) Micrograph of ToF-SIMS analysis area on bone fragment. High spatial resolution ToF-SIMS ion images of B) sum of porphyrin peaks (m/z 441, 455, 469 and 483), C) Fe+, D) Ni+, E) sum of PAHs (m/z 77,91, 139, 141, 165), F) Al+, and G) Si+. H) SEM image of same area as in A. I) Zoom-in of area indicated by right white box in H where porphyrins were detected. J) Zoom-in of area indicated by white box in I with microbodies (white arrows). K) Zoom-in of area indicated by left white box in H with whitish non-bony structure (Fig 1E and 1F). L) Zoom-in of area indicated by white box in K showing fibrous structure (white arrow). (PDF) [file pone.0269568.s004.pdf]

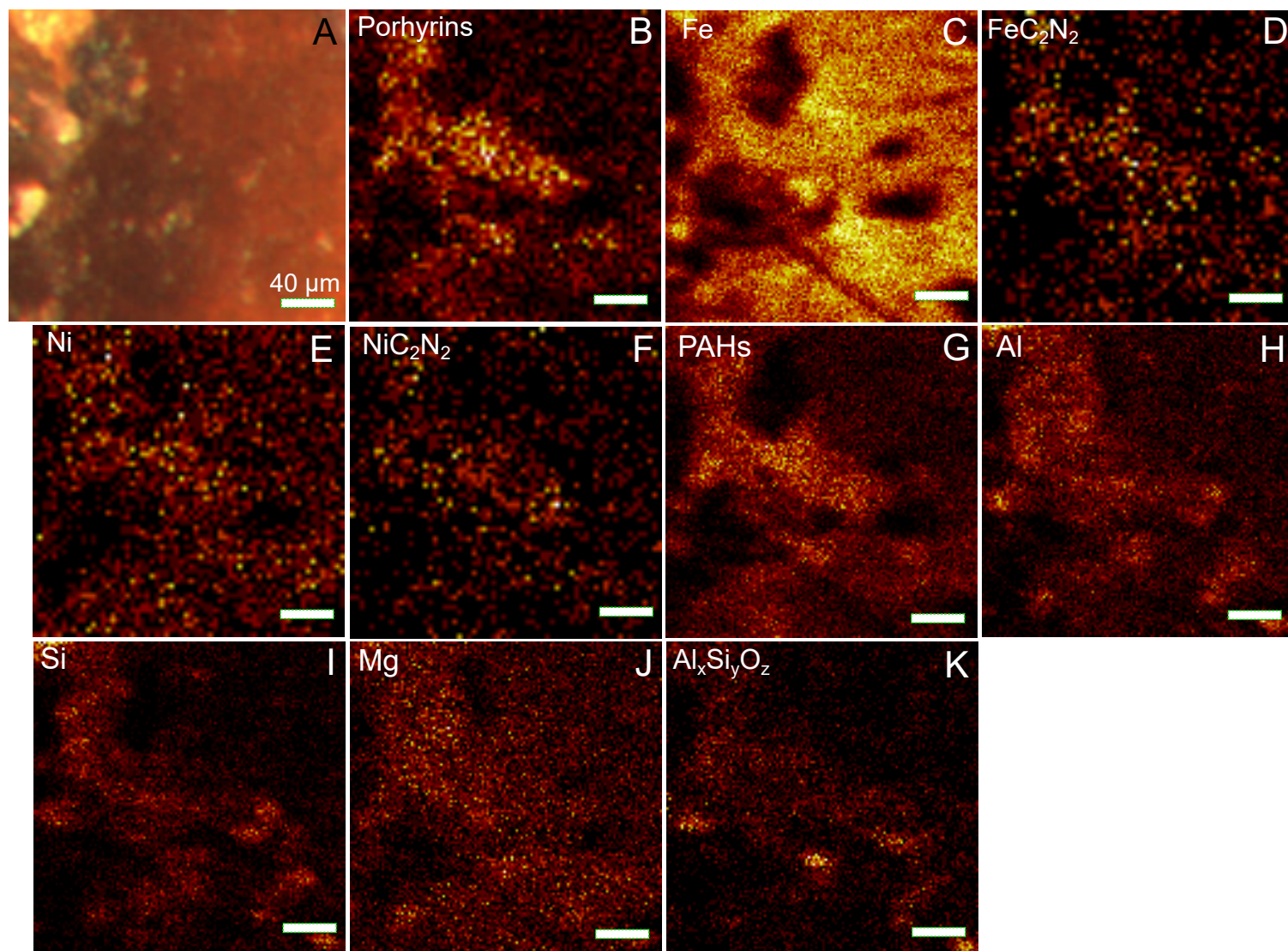

Supplementary Figure 5

Supplement: S5 Fig — A) Micrograph of ToF-SIMS analysis area on ganoid scale 2. High spatial resolution ToF-SIMS ion images of B) sum of porphyrin peaks (m/z 441, 455, 469 and 483) C) Fe+, D) FeC2N2-, E) Ni+, F) NiC2N2-, G) sum of PAHs (m/z 77, 91, 139, 141, 165), H) Al+, I) Si+, K) Mg+ and K) sum of AlSiO4- and AlSi2O6-. (PDF) [file pone.0269568.s005.pdf]

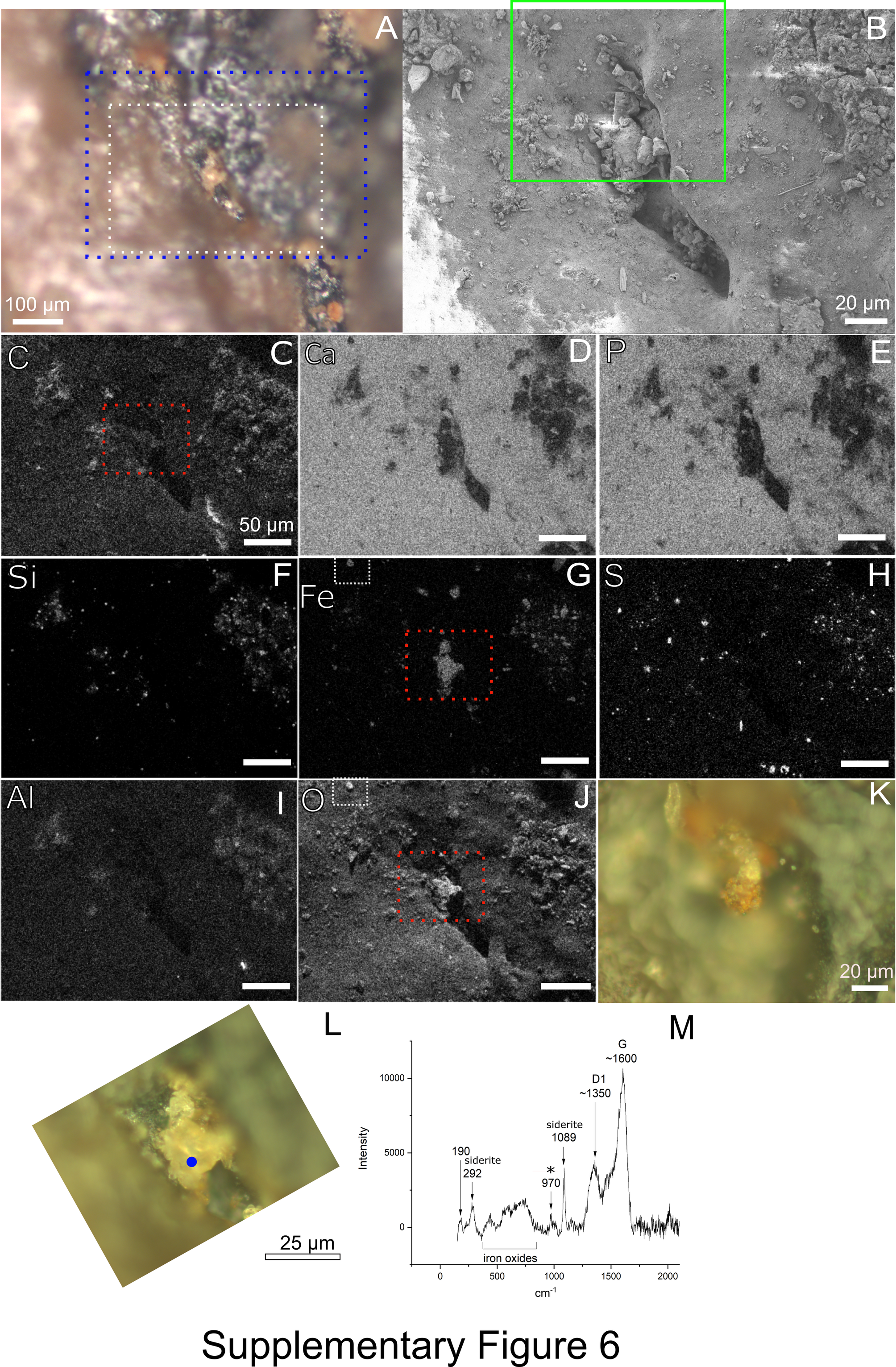

Supplement: S6 Fig — A) Micrograph of area of ToF-SIMS analyses on fish bone fragment. B) SEM image of area indicated by white dotted box in A. Green square indicates area of ToF-SIMS analysis in S4 Fig. C-J) EDX maps of area indicated by blue dotted box in B. K) Micrograph of area indicated by white dotted boxes in G and J where Fe and O co-localize. L) Micrograph of area of red dotted boxes in C, G and J showing area where C, Fe and O co-localize. Blue circle shows area of Raman analysis in M. M) Blue laser Raman spectrum of mineral grain attached to fossil showing presence of siderite (1089 cm-1), organic material (D1 and G) and iron oxides (⁓400–800 cm-1). Peak marked with * can tentatively be assigned to poorly crystalline calcium phosphates. (TIF) [file pone.0269568.s006.tif]

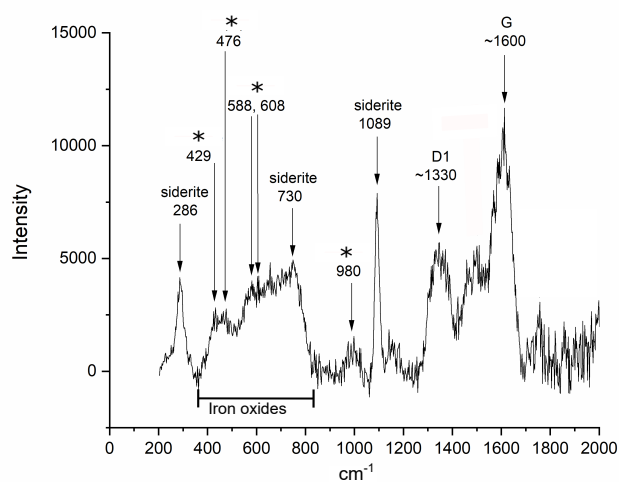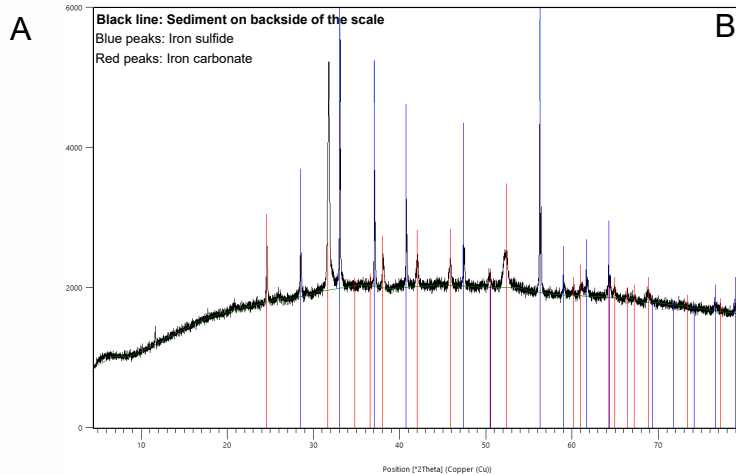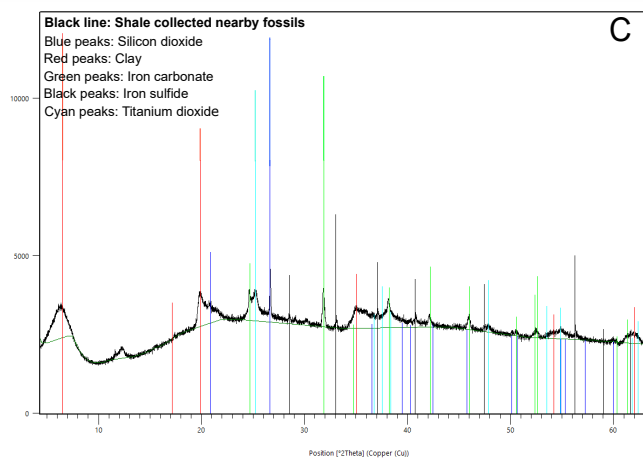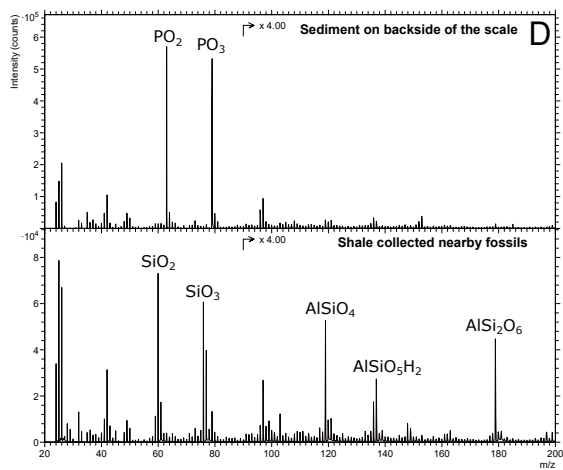

Supplementary Figure 7

Supplement: S7 Fig — A) Blue Raman spectrum of sediment attached to back of ganoid scale 1 showing presence of siderite (1089 cm-1), organic material (D1 and G) and iron oxides (⁓400–800 cm-1). Peaks marked with * can tentatively be assigned to poorly crystalline calcium phosphates. XRD diffractogram of B) sediment attached to back of ganoid scale 1 and C) shale collected nearby (S1 Fig). D) ToF-SIMS spectra of sediment attached to back of ganoid scale 1 (top) and shale collected nearby (bottom). Notice the difference in mineralogy of sediment directly attached to fossil, mainly siderite, calcium phosphates, iron oxides and pyrite versus shale collected further away which is enriched in silicates. (PDF) [file pone.0269568.s007.pdf]

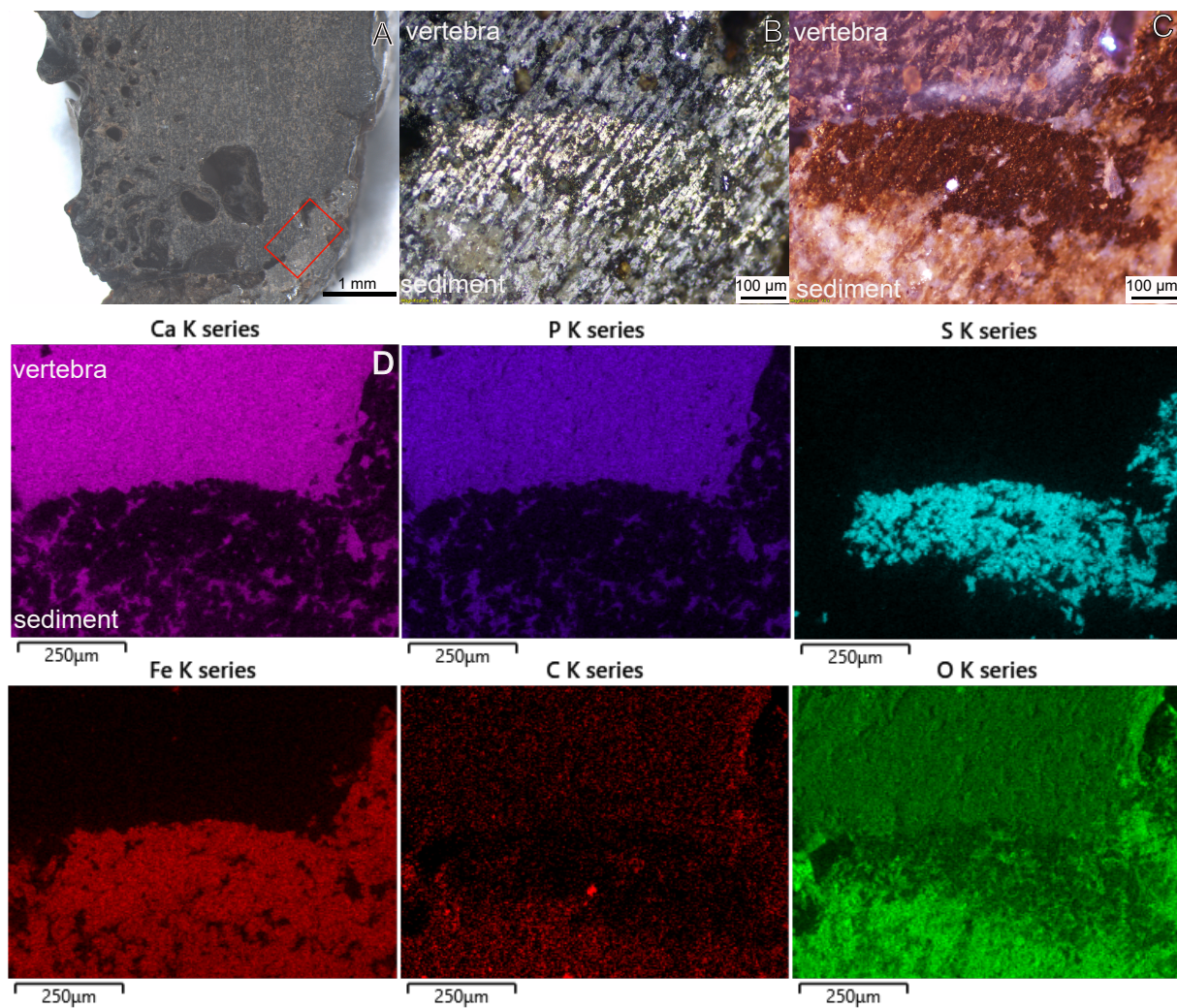

Supplementary Figure 8

Supplement: S8 Fig — A) Micrograph of fossil/sediment interface on fossil vertebra (S1C Fig). Red box indicates zoom-in B). C) Fluorescence micrograph of same area as B. D) EDX maps of same area as A-B showing Ca, P, S, Fe, C and O. The data indicates presence of pyrite, calcium phosphates, iron oxides and siderite in sediment. Note that the area of EDX maps is slightly shifted compared with micrographs in A-B. (PDF) [file pone.0269568.s008.pdf]
