# Supplementary material for: Detection of porphyrins in vertebrate fossils from the Messel and implications for organic preservation in the fossil record
Source: PLoS One. 2022 Jun 29;17(6):e0269568. doi: 10.1371/journal.pone.0269568 (PMC9242450; doi:10.1371/journal.pone.0269568)
Supplement: S1 Table — (DOCX) [file pone.0269568.s009.docx]

| Assignment | Observed ions in fish fossils (+ and -) | Tentative formula |
| --- | --- | --- |
| Heme fragments | - |  |
|  | 497.08 | [C_30_H_25_FeN_4_]^+^ |
|  | 485.11 | [C_29_H_23_NiN_4_]^+^ |
|  | 483.09 | [C_29_H_23_FeN_4_]^+^ |
|  | 471.08 | [C_28_H_21_NiN_4_]+ |
|  | 469.08 | [C_28_H_21_FeN_4_]^+^ |
|  | 457.06 | [C_27_H_19_NiN_4_]+ |
|  | 455.07 | [C_27_H_19_FeN_4_]^+^ |
|  | 441.05 | [C_26_H_17_FeN_4_]^+^ |
|  |  |  |
|  | 499.10 | [C_30_H_27_FeN_4_]^-^ |
|  | 485.10 | [C_29_H_23_FeN_4_]^-^ |
|  |  |  |
| PAHs | 77.04 | [C_6_H_5_]^+^ |
|  | 91.05 | [C_7_H7]^+^ |
|  | 115.05 | [C_9_H7]^+^ |
|  | 128.05 | [C_10_H_8_]^+^ |
|  | 139.05 | [C_11_H_7_]^+^ |
|  | 141.06 | [C_11_H_9_]^+^ |
|  | 165.07 | [C_13_H_9_]^+^ |
|  |  |  |
| Fe | 55.93 | Fe^+^ |
| FeC_2_N_2_ | 107.94 | [FeC_2_N_2_]^-^ |
| Ni | 57.94 | Ni^+^ |
| NiC_2_N_2_ | 109.94 | [NiC_2_N_2_]^-^ |
| ^60^NiC_2_N_2_ | 111.94 | [^60^NiC_2_N_2_]^-^ |
|  |  |  |
| Al | 26.98 | Al^+^ |
| Si | 27.97 | Si^+^ |
| Al_x_Si_y_Oz |  |  |
|  | 118.94 | [AlSiO_4_]^-^ |
|  | 178.90 | [AlSi_2_O_6_]^-^ |

**S1 Table**
