# Supplementary material for: Detection of porphyrins in vertebrate fossils from the Messel and implications for organic preservation in the fossil record
Source: PLoS One. 2022 Jun 29;17(6):e0269568. doi: 10.1371/journal.pone.0269568 (PMC9242450; doi:10.1371/journal.pone.0269568)
Supplement: S2 Table — (DOCX) [file pone.0269568.s010.docx]

| **Element (weight %)** | **Point spectrum 1 inside grey-brown film on ganoid scale 1** | **Point spectrum 2 inside grey-brown film on ganoid scale 1** | **Point spectrum outside grey-brown film on ganoid scale 1** | **Porphyrin-rich area on bone** | **Sediment attached to scale** | **Sediment attached to scale** | **Nearby collected shale** | **Nearby collected shale** |
| --- | --- | --- | --- | --- | --- | --- | --- | --- |
| **C K** | 15.55 | 13.96 | 11.39 | 18.56 | 24.74 | 32.35 | 34.30 | 56.11 |
| **O K** | 49.43 | 42.54 | 42.52 | 41.19 | 40.20 | 36.24 | 40.17 | 29.05 |
| **Ca K** | 16.21 | 25.07 | 27.05 | 23.42 | 16.70 | 13.90 | 0.74 | 0.65 |
| **P K** | 9.67 | 14.31 | 15.31 | 11.87 | 9.71 | 7.91 | 0.23 | 0.14 |
| **Fe K** | 2.01 | 1.18 | 1.13 | 2.99 | 5.84 | 7.46 | 4.18 | 2.73 |
| **Si K** | 3.99 | 0.36 | 0.22 | 0.62 | 0.93 | 0.65 | 12.26 | 6.36 |
| **Al K** | 1.90 | 0.27 | 0.17 | 0.43 | 0.38 | 0.18 | 5.68 | 2.84 |
| **S K** | 0.25 | 0.21 | 0.12 | 0.41 | 0.91 | 0.69 | 0.72 | 1.08 |
| **Mg K** | 0.28 | 0.13 | 0.07 | 0 | 0.20 | 0.25 | 0.54 | 0.33 |
| **Mn K** | 0 | 0 | 0 | 0.27 | 0.20 | 0.21 | 0.04 | 0.07 |
| **Ti K** | 0.40 | 0.02 | 0.06 | 0 | 0 | 0 | 0.97 | 0.61 |
| **Na K** | 0.30 | 0.51 | 0.54 | 0.24 | 0.19 | 0.17 | 0.02 | 0.00 |
| **F K** | 0 | 1.44 | 1.42 | 0 | 0 | 0 | 0 | 0 |
| **Si/Al ratio** | 2.1 | 1.33 | 1.29 | 1.44 | 2.45 | 3.61 | 2.16 | 2.24 |

Note: Locations of point spectra on ganoid scale 1 are indicated with red dots in Fig 5C. Data from bone come from area in S6C-J.

**S2 Table**
